# VEL-dependent polymerization maintains the chromatin association of Polycomb proteins for the switch to epigenetic silencing

**DOI:** 10.1016/j.molcel.2025.08.002

**Published:** 2025-08-25

**Authors:** Anna Schulten, Geng-Jen Jang, Alex Payne-Dwyer, Marc Fiedler, Mathias L. Nielsen, Eduardo Mateo-Bonmatí, Mariann Bienz, Mark C. Leake, Caroline Dean

**Affiliations:** 1 Department of Cell and Developmental Biology, John Innes Centre, Norwich NR4 7UH, UK; 2 School of Physics, Engineering and Technology, University of York, York YO10 5DD, UK; 3 Department of Biology, University of York, York YO10 5DD, UK; 4 Medical Research Council Laboratory of Molecular Biology, Cambridge CB2 0QH, UK

## Abstract

Multivalent protein-chromatin interactions facilitated by higher-order protein assemblies are emerging as a crucial theme in eukaryotic gene regulation. However, understanding the underlying mechanisms in their functional context remains challenging. *Arabidopsis* VEL proteins assemble biomolecular condensates by head-to-tail polymerization. Here, we dissect the role of VEL polymerization domains in conferring the epigenetic switch to Polycomb repressive complex 2 (PRC2) silencing at *Arabidopsis FLOWERING LOCUS C* (*FLC*). We show that VIN3 VEL polymerization produces higher-order nuclear VIN3 assemblies *in vivo*, which promote multivalent chromatin association and efficient H3K27me3 nucleation. VRN5 VEL polymerization, however, is not required unless a third homolog VEL1 is absent. The VRN5 VEL domain has different polymerization properties and is functionally unable to replace VIN3 VEL, but it is required to physically connect VIN3 with PRC2. This work reveals the combinatorial roles of VEL polymerization domains in maintaining the chromatin association of Polycomb proteins for the switch to epigenetic silencing.

## Graphical abstract

**Graphical abstract:**
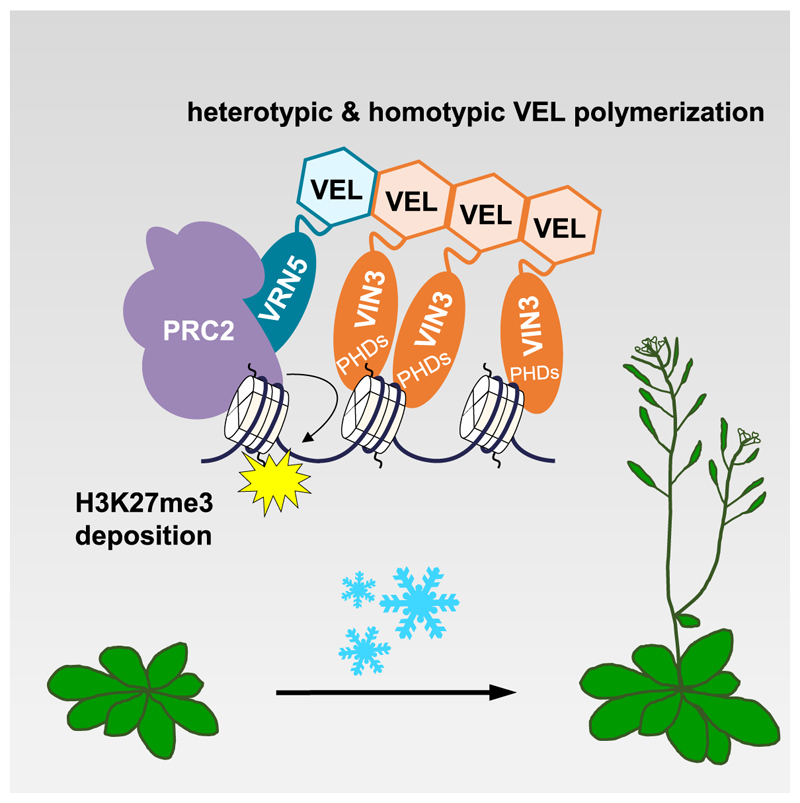

## Introduction

The clustering of macromolecules is an important mechanism in the regulation of cellular processes. Such high local concentration of these macromolecules, often described as biomolecular condensation, enables efficient interactions between them. As proteins cluster into assemblies, they can present multiple binding sites simultaneously and promote the formation of cooperative multivalent interactions based on individually weak affinities (“functional affinity” or avidity).^1–3^ In recent years, combinatorial protein inputs from higher-order protein assemblies have emerged as a general theme for orchestrating transcriptional output in eukaryotic gene regulation, capable of buffering against noisy signals and enhancing target specificity. Experiments with transcription factors (TFs) in yeast serve as an example of the physiological relevance of such mechanisms: synthetic TFs with high affinity to *cis*-regulatory motifs imposed a fitness burden that was relieved by weakly binding TFs cooperating in assemblies.^4^

The mechanisms that facilitate higher-order assembly of proteins include intrinsically disordered regions (IDRs)^5^ and polymerization domains. The latter enable structurally more defined assemblies formed by the self-association of proteins via two distinct interfaces—referred to as head and tail—to assemble dynamic and reversible head-to-tail polymers.^6^ Polymerization has been shown to be functionally important for a number of DNA-binding TFs and proteins that associate with histone-modifying complexes of the Polycomb group.^7–12^

**Figure 1:**
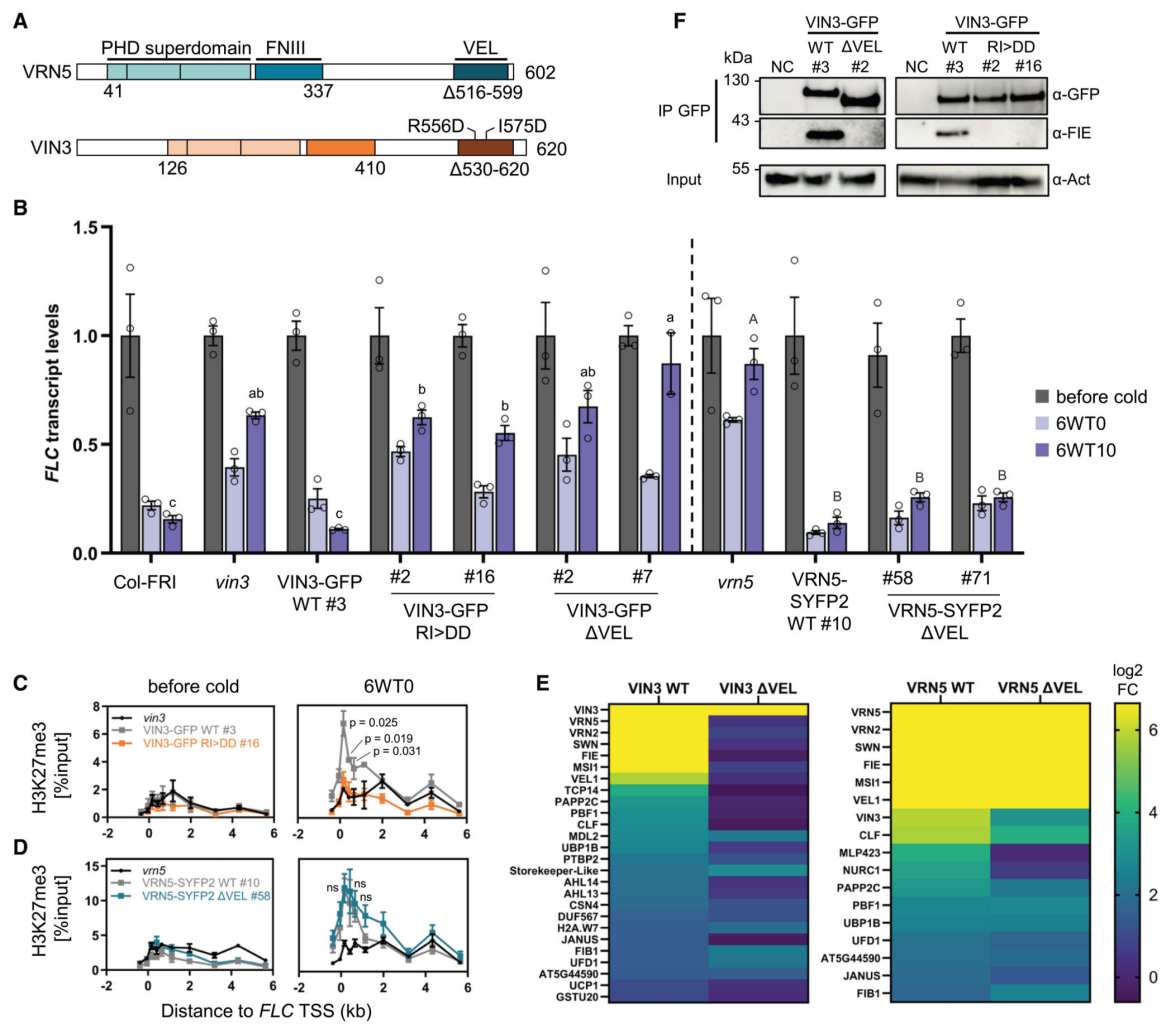
Mutations in VIN3 and VRN5 VEL domains confer contrasting phenotypes (A) Domain architecture of VEL proteins VIN3 and VRN5. Tripartite PHD superdomain: a zinc finger, an atypical PHD domain, and a four-helix bundle. Above: R556D and I575D indicate polymerization-blocking point mutations in VIN3 VEL; below: amino acids deleted in VIN3-GFP ΔVEL and VRN5-SYFP2 ΔVEL. (B) RT-qPCR assays of *FLC* transcripts during a vernalization time course; before cold, after 6-week cold exposure (6WT0), or 10 days post-cold (6WT10). Data are relative to the geometric mean of *UBC*/*PP2A*, normalized to *FLC* before cold. Error bars represent standard deviations (*n* = 3 biological replicates). Different letters denote significant differences (*p* < 0.05) between means based on ANOVA with post hoc Tukey’s HSD. (C and D) H3K27me3 ChIP across the *FLC* locus before cold and after 6-week cold, relative to input, in (C) VIN3 or (D) VRN5 VEL domain mutant lines. TSS: transcriptional start site. Error bars represent SEM (*n* = 3 biological replicates). Differences between WT and VEL domain mutant lines were tested with two-tailed t test for primer pairs covering the nucleation region. (E) Heatmap from IP-MS samples showing nuclear proteins co-precipitating with VIN3 and VRN5 baits in vernalized seedlings. Log2 fold change (FC) is in comparison to non-transgenic Col-FRI (adj. *p* ≤ 0.05 for wild-type proteins, *n* = 3 biological replicates). (F) Immunoblots of α-GFP immunoprecipitates from vernalized plants with VIN3-GFP transgenes, probed with α-FIE (PRC2 core). Non-transgenic Col-FRI was used as negative control (NC). Blots shown are representative of three replicates.

This group includes *Arabidopsis thaliana* proteins of the VEL family (VIN3, VRN5[/VIL1], and VEL1[/VIL2]). These are accessory proteins of the widely conserved Polycomb repressive complex 2 (PRC2) whose activity is essential for the deposition of H3K27me3—one of the hallmarks of mitotically heritable gene silencing. The VEL proteins undergo homotypic and heterotypic interactions via their VEL domain, a head-to-tail polymerization domain that is conserved through the green lineage.^11^ The spontaneous homo-polymerization of VIN3 and VEL1 via their VEL domains drives their assembly into dynamic biomolecular condensates when expressed in heterologous systems.^11^ The VEL proteins also contain a tripartite plant homeodomain (PHD) superdomain and a fibronectin type III (FNIII) domain^13,14^ (Figure 1A). This shared domain architecture suggested similar functionality, with one protein being cold-induced (VIN3) and the others (VRN5, VEL1) constitutively expressed.^15^ However, recent structural analysis has revealed that specifically in VRN5, there is a close packing of the central PHD superdomain and FNIII domain, and this mediates its interaction with a PRC2 core complex with the core subunit VRN2 (one of three *Arabidopsis* homologs of mammalian SUZ12).^16,17^ By contrast, VIN3 has a more open conformation of these domains and depends on VRN5 to interact with PRC2.^17^

The genome-wide chromatin association of VEL proteins suggests a widespread role in PRC2 silencing of many loci in the *Arabidopsis* genome.^17^ Genetic analyses showed that VIN3 and VRN5, but not VEL1, are essential for the epigenetic silencing of the floral repressor gene *FLOWERING LOCUS C* (*FLC*) during the winter cold, a process called vernalization.^15,18–20^ At *FLC*, Polycomb silencing initiates via coldinduced nucleation of H3K27me3 over a small number of nucleosomes around the first exon-intron boundary of *FLC*. This confers metastable epigenetic silencing that holds the silent state for tens of cell cycles.^21^ The metastable state is converted into a long-term epigenetically silenced state through the spreading of H3K27me3 across the *FLC* gene body, which occurs when the plants start to grow more rapidly following the return to warmer temperatures occurring in spring.^22^ Nucleation, which is strongly reduced in *vin3, vrn5*, and *vrn2* single mutants, can be separated from the stable spread H3K27me3 state in mutants defective in one of the PRC2 methyltransferases genes, *CURLY LEAF* (*CLF*) or *LIKE HETEROCHROMATIN PROTEIN 1*, required to maintain long-term silencing.^23^ Studies in these mutants have shown that VEL proteins promote the stochastic PRC2-mediated nucleation of individual *FLC* alleles, whereby the fraction of nucleated *FLC* loci increases over prolonged cold of winter.^24^ In other words, the slow accumulation of H3K27me3 at the 3–4 nucleosome nucleation region is the result of a low probability digital ON/OFF switch, which occurs independently at each allele.^22,25^

Mathematical modeling of epigenetic state switching and memory at *FLC* had predicted the requirement of additional protein memory storage elements that positively feedback to reinforce themselves; for example, molecular assemblies that are maintained in sufficiently high numbers to overcome the nucleation threshold and that may persist at the locus even through the nucleosome perturbations that occur during DNA replication.^21^ Nuclear assemblies of VEL proteins increase in stoichiometry during vernalization,^26^ so that VEL-dependent protein polymerization and condensate formation could provide such a mechanism. This system thus constitutes an excellent tool to dissect the functional relevance of higher-order protein assemblies *in vivo*. Here, we therefore experimentally investigated the roles of VEL polymerization domains in the switch to the epigenetically silenced state.

## Results

### Contrasting PRC2-related phenotypes in VIN3 and VRN5 VEL domain mutants

We had previously shown that stable Arabidopsis lines expressing VIN3-GFP with single amino acid polymerization-blocking point mutations located in the head and tail of the VEL domain (R556D/I575D [RI>DD], Figure 1A) fail to rescue impaired *FLC* silencing in the *vin3* mutant.^11^ This pointed to the importance of polymerization in *FLC* silencing. Given that polymerization of the VEL proteins is concentration dependent,^11^ we chose to directly compare the effect of VIN3-GFP wild-type (WT) and RI>DD transgenes when expressed at endogenous levels. Two homozygous single-insertion lines with protein levels equal to a single-insertion VIN3-GFP WT line were selected from a larger T2 generation, all displaying the same non-complementation phenotype (Figure S1). These VIN3-GFP RI>DD lines mirrored the impaired *FLC* shutdown during cold and the resulting high post-cold *FLC* levels observed for lines with a deletion of the entire VEL domain in VIN3-GFP (Figures 1B and S1). Besides, H3K27me3 failed to accumulate at the *FLC* nucleation region in VIN3-GFP RI>DD (Figure 1C). Because the RI>DD mutation disrupts VIN3 polymerization with minimal impact on the rest of the protein, we conclude that the polymerization of the VIN3 VEL domain is required to promote PRC2-mediated deposition of H3K27me3 in the *FLC* nucleation region.

Our prediction from the heterotypic interactions observed between VIN3 and VRN5 was that the VEL domain would be required for silencing by mediating the VRN5-dependent interaction between VIN3 and PRC2.^15,17^ However, independent homozygous stably transformed Arabidopsis VRN5-SYFP2 ΔVEL lines with single transgene insertions in a *vrn5* mutant background (Figures 1A and S2) showed an unexpected result; *FLC* was fully silenced in VRN5-SYFP2 ΔVEL like in a *vrn5* rescue line expressing VRN5-SYFP2 WT (Figure 1B). In agreement, the impaired deposition of H3K27me3 observed in the *vrn5* mutant was also rescued in VRN5 ΔVEL plants (Figure 1D).

To further understand the different phenotypes observed for the VEL domain deletions of VIN3 and VRN5 *in vivo*, we determined the interaction partners of VIN3 and VRN5 dependent on the VEL domain. Native GFP co-immunoprecipitation (coIP) followed by mass spectrometry (MS) was undertaken with respective WT and ΔVEL seedlings vernalized for 6 weeks. Both VIN3 and VRN5 WT proteins co-precipitated all components of one of the Arabidopsis PRC2 core complexes (VRN2, FIE, MSI1, SWN, and to a lesser extent CLF), as well as one another and VEL1 as expected (Figure 1E). The deletion of the VIN3 VEL domain resulted in the loss of all PRC2 core subunits and VRN5/VEL1. Probing for the PRC2 subunit FIE with immunoblot analysis after coIP in extracts of vernalized plants confirmed the loss of FIE interaction in both VIN3 ΔVEL and VIN3 RI>DD lines (Figure 1F). Together with our previous result that VIN3 depends on VRN5 for PRC2 interaction,^17^ this suggests that VEL-mediated interaction between VIN3 and VRN5 is required for VIN3 to associate with the PRC2 core complex *in vivo*. In contrast, the interaction with PRC2 was maintained in VRN5 ΔVEL plants (Figure 1E). This is in accordance with our previous results from heterologous coIPs in mammalian cells, which mapped the compact conformation of the PHD superdomain and the FNIII of VRN5 but not the VEL domain to the interface for PRC2 interaction.^17^ Surprisingly, the interaction of VRN5 ΔVEL with VEL1 was also maintained. VEL1 interacts specifically with the PRC2 core subunit MSI1, which may explain the association with VRN5 ΔVEL.^17^

### Role of VEL domain in nuclear assembly formation of VIN3 and VRN5

The protein assemblies predicted to be involved in Polycomb epigenetic switching can in principle be achieved by VEL polymerization-based assemblies or by multimeric assemblies mediated by other interactors such as PRC2 (or combinations thereof). We therefore used the stable *Arabidopsis* transgenic VIN3-GFP and VRN5-SYFP2 lines to investigate the effects of VEL domain disruptions on the stoichiometry of both VIN3 and VRN5 in molecular assemblies *in vivo*. We performed single-particle tracking of the fluorescently tagged proteins with SlimVar microscopy, which uses an oblique illumination for rapid, enhanced imaging contrast, enabling single-molecule detection sensitivity in intact root tips.^26^ Stepwise photobleaching is employed as a calibration to determine the number of tagged molecules in detected fluorescent particles.^27^ The stoichiometry is the ratio of the particle’s initial intensity compared with the characteristic brightness of a single fluorescent protein (see examples in Figures S3A and S3B).

**Figure 2:**
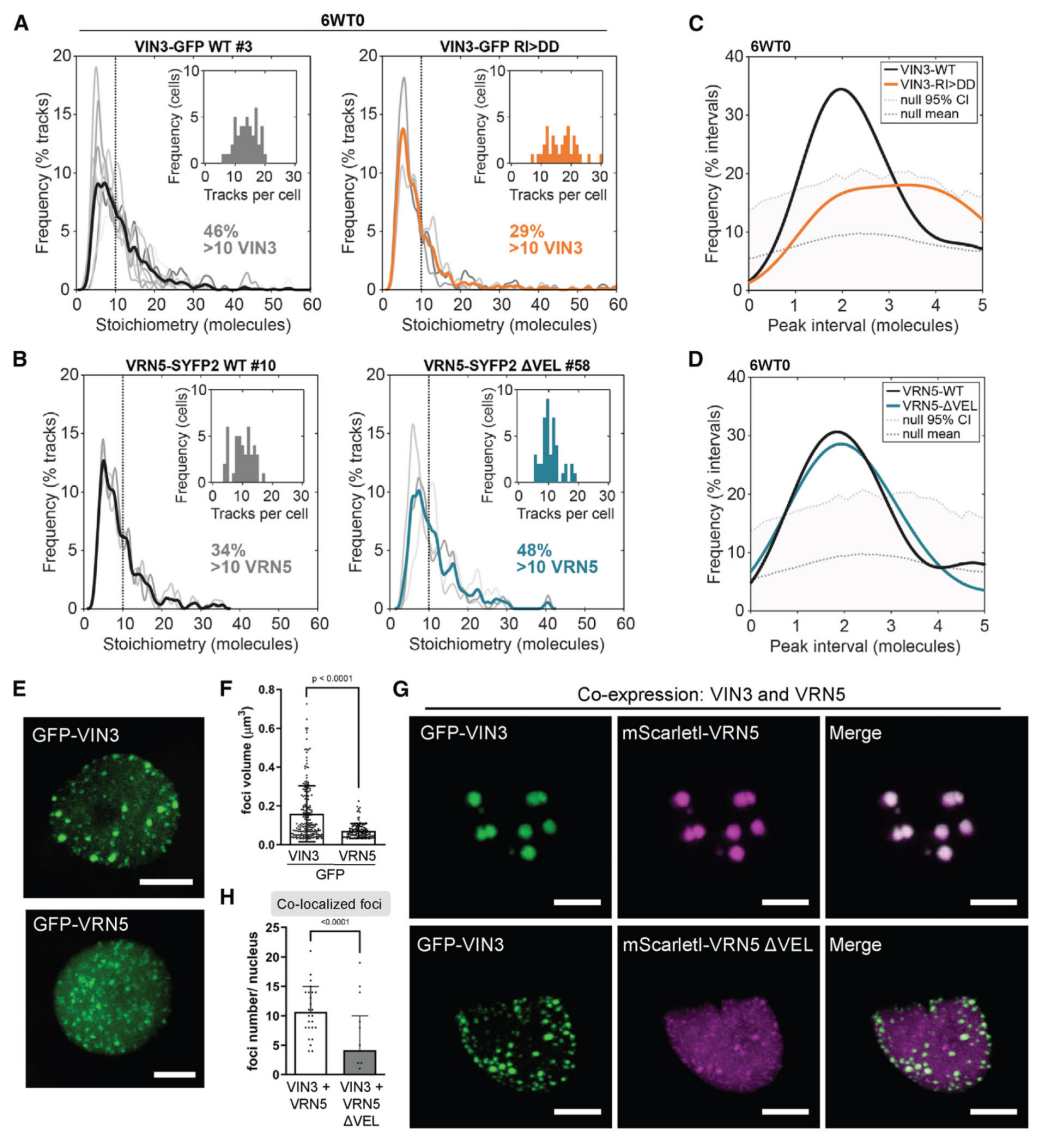
*In vivo* VEL-dependent VIN3 and VRN5 assemblies in stable *Arabidopsis* transgenic lines and after transient heterologous expression (A and B) Stoichiometry (molecule number) distributions of SlimVar tracked assemblies of (A) VIN3-GFP WT and VIN3-GFP RI>DD (WT larger than RI>DD by Brunner Munzel test: *p* < 0.0001) and (B) VRN5-SYFP2 WT and VRN5-SYFP2 ΔVEL (ΔVEL larger than WT by Brunner Munzel test: *p* < 0.0001) in root nuclei of seedlings vernalized for 6 weeks. Individual replicates (*n* = 3–8 experiments, with 9–18 nuclei each) in gray, and bold line indicates mean distribution. Insets show the frequency of tracks per cell, data collection statistics: Figure S3. (C and D) Distributions of intervals between nearest-neighbor stoichiometry peaks of tracked assemblies in (A and B). Upper dotted line indicates the null 95% confidence interval determined from simulations of random aperiodic stoichiometry, distributions that fall below are consistent with the null hypthesis.^23^ (E and G) Representative confocal images of epidermal leaf cell nuclei in *N. benthamiana*, (E) transiently expressing GFP-VIN3 or GFP-VRN5 and (G) transiently co-expressing GFP-VIN3 (green) and mScarletI-VRN5 (magenta) wild type or mutant as indicated; scale bars, 5 μm. (F) Quantification of foci volume in (E); error bars represent standard deviation (*n* = 199 for VIN3, *n* = 135 for VRN5). (H) Quantification of co-localized foci per nucleus in (G); error bars represent standard deviation (*n* = 20–25 nuclei). *p* values indicate statistically significant differences based on two-tailed t test. See also Figures S3 and S4.

In roots of seedlings vernalized for 6 weeks, we observed that the VIN3-GFP RI>DD mutations resulted in VIN3 assemblies with a lower number of molecules; only 29% of tracks corresponded to assemblies larger than 10 molecules, compared with 46% in VIN3-GFP WT (Figure 2A). This suggests that VEL-mediated polymerization contributes to the formation of VIN3 assemblies in plant cells at endogenous protein concentration. For VRN5-SYFP2 ΔVEL, we observed an increase in assembly stoichiometry, in comparison with that of the WT, in seedlings vernalized for 6 weeks (Figure 2B, for other time points see Figures S3C and S3D), in contrast to the decrease expected if the assemblies were VEL-mediated. We then conducted a periodicity analysis to assess whether consistent intervals existed between nearest-neighbor peaks in the assembly stoichiometry distributions.^26^ The distributions of WT VEL proteins show intervals of two molecules consistent with dimeric subunits of VEL proteins within the assemblies. This pattern was disrupted in VIN3-GFP RI>DD (Figure 2C) but generally maintained for VRN5-SYFP2 ΔVEL (Figure 2D). Notably, we have previously observed VIN3 VEL domain crystals composed of protofilaments with a dimeric unit; the result of a mutual domain swapping of VEL domain helices between two VIN3 monomers.^11^ It remains to be determined whether PRC2 might also contribute to VIN3 assembly formation during vernalization. Given the direct interaction between VRN5 and PRC2, we speculate that VEL-independent dimeric periodicity of VRN5 may be a result of the reported ability of PRC2 to undergo dimer formation.^28–30^

Because we observed no reduction in the size of VRN5 assemblies and no effect on *FLC* silencing in stable *Arabidopsis* lines carrying VRN5 ΔVEL, we investigated the properties of the VRN5 VEL domain. VIN3 polymerization results in the formation of biomolecular condensates,^11^ so we assayed VRN5 condensate formation after expression in leaf epidermal cells of *Nicotiana benthamiana* (*N. benthamiana*) via agroinfiltration. This widely used transient assay is excellent for the characterization of protein properties but has the caveat that the proteins are overexpressed relative to endogenous levels.

In comparison with the VEL- and polymerization-dependent discrete condensates formed by GFP-VIN3,^11^ GFP-VRN5 formed significantly smaller condensates, with more VRN5 protein diffusely distributed throughout the nucleus (Figures 2E, 2F, S4A–S4C). Foci observed after tagging VRN5 with mScarletI also support the interpretation that VRN5 foci are overall smaller than the foci observed for VIN3, with the deletion of the VRN5 VEL domain abolishing the formation of any discrete VRN5-mScarletI condensates (Figures S4D and S4E). Upon expression in HeLa cells, GFP-VRN5 previously also appeared diffuse.^11^ These observations are consistent with VRN5 having a lower propensity to concentrate into condensates in a VEL-dependent manner. In agreement with our *in vivo* coIP results, we observed the recruitment of mScarletI-VRN5 into GFP-VIN3 to form large condensates in the *N. benthamiana* system, a co-localization that was dependent on the VRN5 VEL domain (Figures 2G, 2H, and S4F). Thus, we conclude that the VRN5 VEL domain has some ability to homopolymerize and heteropolymerize, but its properties appear different to the VEL domain of VIN3.

### VRN5 VEL domain is not functionally equivalent to VIN3 VEL

**Figure 3:**
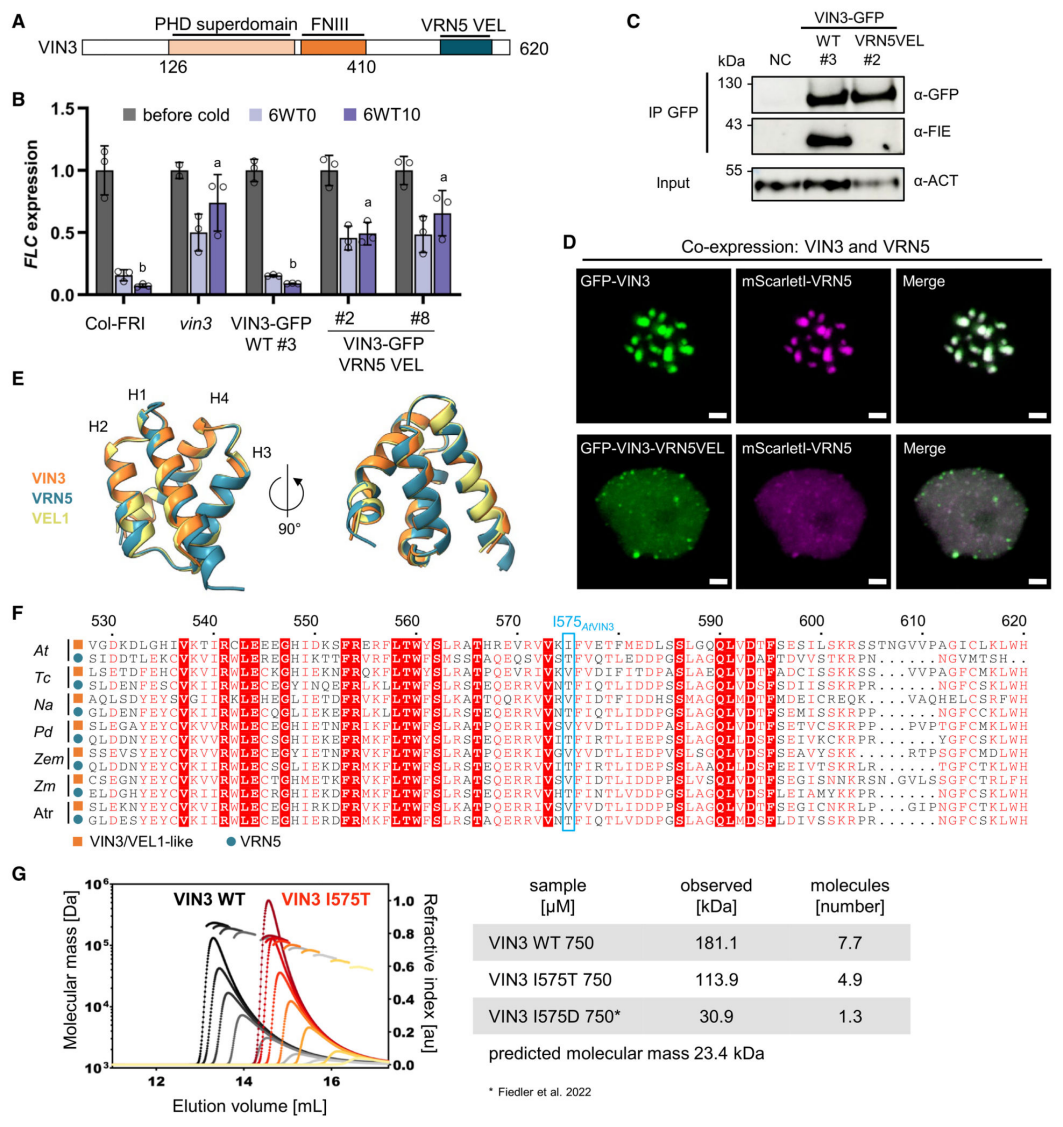
VRN5 VEL cannot replace VIN3 VEL function *in planta* (A) VIN3-GFP VRN5 VEL chimera construct transformed into the *vin3* mutant. (B) RT-qPCR assays of *FLC* transcripts during a vernalization time course; before cold, after 6-week cold exposure (6WT0), or 10 days post-cold (6WT10). Data are relative to the geometric mean of *UBC*/*PP2A*, normalized to *FLC* before cold. Error bars represent standard deviations (*n* = 3 biological replicates). Different lowercase letters denote significant differences (*p* < 0.05) between means based on ANOVA with post hoc Tukey’s HSD. (C) Immunoblots of α-GFP immunoprecipitates from vernalized plants with indicated VIN3-GFP transgenes, probed with α-FIE (PRC2 core). Non-transgenic Col-FRI was used as NC. Blots shown are representative of three replicates. (D) Representative confocal images of epidermal leaf cell nuclei in *N. benthamiana*, transiently co-expressing GFP-VIN3 or GFP-VIN3 VRN5 VEL (green) and mScarletI-VRN5 (magenta); scale bars, 2 μm. See also Figure S6. (E) Superpositions of VEL domains of *Arabidopsis* VRN5_515-592_ (teal), VIN3_529-601_ (orange), and VEL1_618-690_ (yellow), as predicted by Alphafold. VIN3 and VEL1 AF predictions superpose closely with experimentally determined structures.^11^ (F) Amino acid sequence conservation of VEL domains of VRN5 (teal, defined by DLNxxxVPDLN motif in the linker region between the FNIII and VEL domains^14^) and VIN3/VEL1 orthologs (orange) throughout the angiosperm lineage. Blue borders highlight the amino acids at the position corresponding to I575_AtVIN3_. *At*: *Arabidopsis thaliana, Tc*: *Theobroma cacao, na*: *Nicotiana attenuata, Pd*: *Phoenix dactylifera, Zem*: *Zea mays, Zm*: *Zostera marina, Atr*: *Amborella trichopoda*. See Figure S7 for full-length protein alignment. (G) SEC-MALS of purified WT (gray to black) or I575T mutant (yellow to red) Lip-VIN3_VEL_ (residues 500–603) at increasing concentrations from right (50 μM) to left (1250 μM); curves: elution profiles (void volume of column at 8 mL); line traces: molar masses as derived from MALS; these are specified in the neighboring table and also indicate numbers of molecules per oligomer at a concentration of 750 μM (note that data for VIN3 I575D are reproduced from Fiedler et al.^11^).

To further examine the properties of the VRN5 VEL domain *in vivo*, we tested whether VRN5 VEL can functionally replace VIN3 VEL in *FLC* silencing. A VIN3-GFP construct, in which the VIN3 VEL domain was replaced with the VRN5 VEL domain (Figure 3A), was transformed into the *vin3* mutant. *FLC* silencing during vernalization remained impaired in two independent homozygous lines as well as in multiple other lines tested in the segregating T2 generation (Figures 3B, S5A, and S5B). This suggests that the VRN5 and VIN3 VEL domains are not functionally equivalent. With equal VIN3 protein expression, coIP of the PRC2 subunit FIE was nearly undetectable in the VIN3-GFP VRN5VEL line, compared with VIN3-GFP WT (Figure 3C), most likely caused by inefficient interaction with endogenous VRN5. To further test this, we performed native GFP coIP followed by MS with VIN3-GFP WT and VIN3-GFP VRN5VEL in seedlings vernalized for 6 weeks and found reduced co-enrichment of VRN5 and PRC2 core subunits in the VIN3-GFP VRN5VEL line (Figure S5C). Equally, the heterologous co-expression of GFP-VIN3 VRN5VEL with mScarletI-VRN5 in these cells did not result in the formation of the large co-localized condensates that are observed upon co-expression of the WT proteins (Figures 3D and S6).

The experimentally determined structures of VIN3 and VEL1 VEL domains align closely with the Alphafold (AF) structure predictions,^11^ and comparing the latter with the AF prediction for the VRN5 VEL domain again shows a close superimposition (Figure 3E). Thus, no major structural differences are predicted to underpin the functional differences between the VEL domains. Next, we decided to compare the amino acid conservation in VEL domains of VIN3 and VRN5 homologs in angiosperm plants (Figure 3F; see also Figure S7). Among the amino acid residues that consistently differ between VIN3 and VRN5, a particularly interesting one is the amino acid residue I575_VIN3_(I664_VEL1_). In the VIN3 VEL tail interface, this engages in hydrophobic or electrostatic interactions with two basic residues in the complementary head surface to mediate polymerization; the mutation I575D (as mutated in VIN3 RI>DD) prevents this polymerization. A threonine in the corresponding position of *Arabidopsis* VRN5 and other VRN5 orthologs throughout angiosperm plants is a polar hydrophilic rather than a hydrophobic amino acid.^11^ To investigate the functional significance of this, we generated the I575T mutation in recombinant VIN3_VEL_, bearing a lipoyl solubility tag and purified following expression in *Escherichia coli*, to conduct size-exclusion chromatography coupled with multiangle light scattering (SEC-MALS). In comparison to WT VIN3_VEL_, polymerization was attenuated by I575T but not blocked (Figure 3G, see comparison with the mutation I575D). This result suggests that specific amino acid differences between VIN3 and VRN5 VEL interfaces contribute to different polymerization properties, consistent with reduced VRN5 condensate formation in *N. benthamiana* cells (Figure 2E).

### VEL polymerization promotes multivalent VIN3 chromatin association

**Figure 4:**
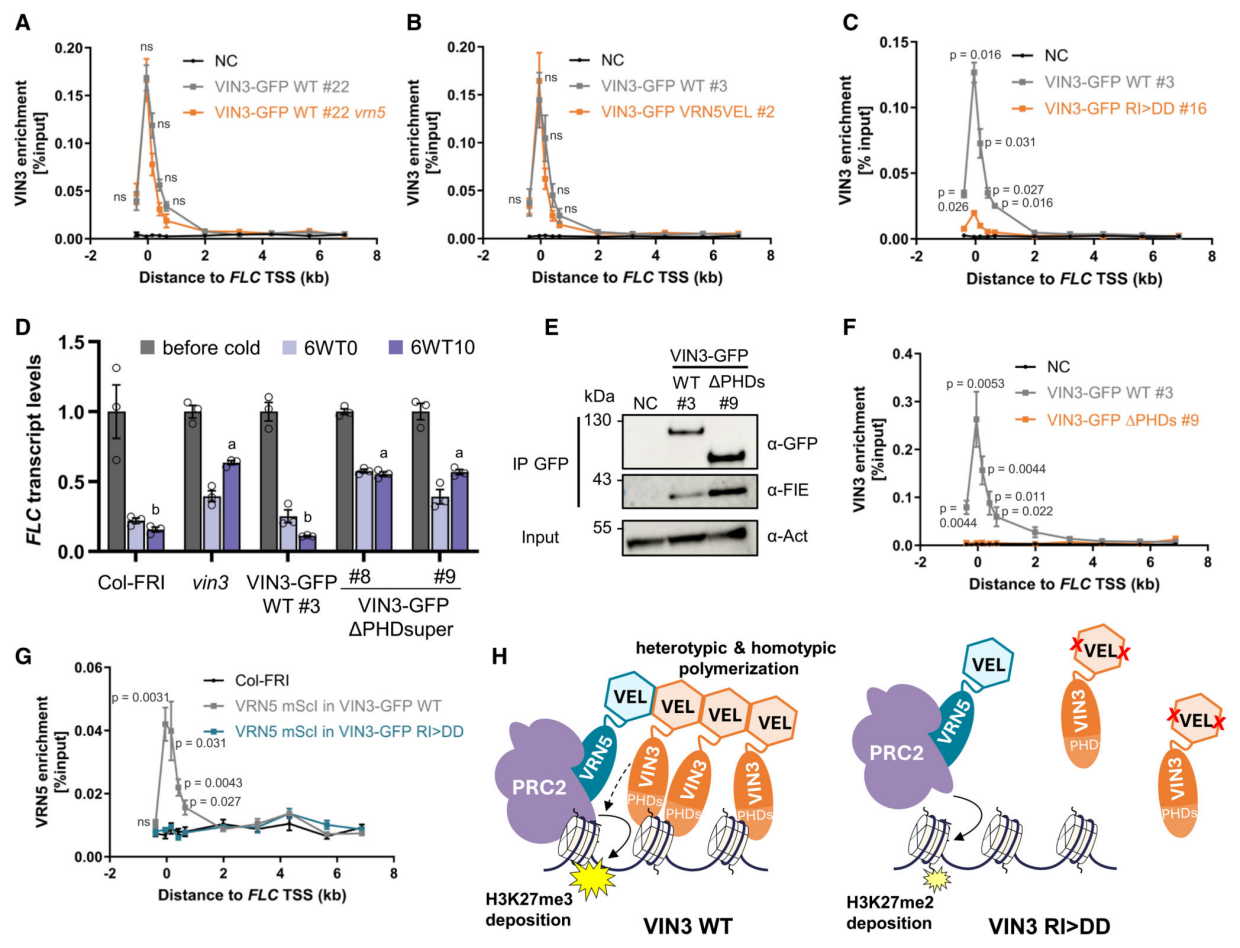
VIN3 chromatin association, promoted by VEL-mediated polymerization and chromatin binding of the PHD superdomain, reinforces VRN5-PRC2 recruitment (A–C and F) ChIP-qPCR showing enrichment of VIN3-GFP (wild type or mutant as indicated) across the *FLC* locus in seedlings vernalized for 6 weeks. Non-transgenic Col-*FRI* plants were used as NC. Data are relative to input control; error bars represent SEM (*n* = 2–4). *p* values indicate statistically significant differences based on two-tailed t test for primer pairs covering the nucleation region. (D) RT-qPCR assays of *FLC* transcripts during a vernalization time course; before cold, after 6-week cold exposure (6WT0), or 10 days post-cold (6WT10). Data are relative to the geometric mean of *UBC*/*PP2A*, normalized to *FLC* before cold. Error bars represent standard deviations (*n* = 3 biological replicates). Different lowercase letters denote significant differences (*p* < 0.05) between means based on ANOVA with post hoc Tukey’s HSD. (E) Immunoblots of α-GFP immunoprecipitates from extracts of vernalized plants bearing the indicated VIN3-GFP transgenes, probed with α-FIE antibody. Non-transgenic Col-FRI was used as a NC. Blots shown are a representative of three replicates. (G) ChIP-qPCR showing enrichment of VRN5-mScarletI in the indicated transgene backgrounds across the *FLC* locus in seedlings vernalized for 6 weeks. Non-transgenic Col-*FRI* plants were used as NC. Data are relative to input control; error bars represent SEM (*n* = 3). *p* values indicate statistically significant differences based on two-tailed t test for primer pairs covering the nucleation region. (H) Schematic model of VIN3 and VRN5 function in PRC2 silencing. Polymerization via the VEL domain promotes VIN3 chromatin association, mediated by emergent multivalent interactions between VIN3 PHD superdomains and chromatin ligands. This reinforces recruitment of VRN5-PRC2 via heterotypic VEL interaction and also promotes H3K27me3 nucleation by other means (dashed arrow, see main text). Note that VIN3/VRN5 stoichiometry drawn here is a representation of the observed differences in VEL domain properties but does not correspond directly to molecule number *in vivo*.

To understand the specific contribution of VIN3 polymerization to *FLC* silencing, we determined whether the loss of interaction between VIN3 and PRC2 observed in the stable VIN3-GFP RI>DD, VIN3-GFP ΔVEL, VIN3-GFP *vrn5*, as well as VIN3-GFP VRN5 VEL lines (Figures 1E, 1F, and 3C) would affect the association of VIN3 with the *FLC* locus. Chromatin immunoprecipitation (ChIP)-qPCR experiments with vernalized seedlings revealed that VIN3-GFP *vrn5* and VIN3-GFP VRN5VEL showed equally high enrichment at *FLC* as VIN3-GFP WT (Figures 4A and 4B), suggesting that the interaction between VIN3 and PRC2 is not per se required for VIN3 chromatin binding. In contrast, we observed a strongly reduced association of VIN3-GFP RI>DD and VIN3-GFP ΔVEL with the *FLC* nucleation region (Figures 4C and S8A). The chromatin association of VRN5 ΔVEL at the *FLC* locus was WT-like in comparison (Figure S8B). We also tested other VIN3 targets, previously identified by ChIP-seq experiments,^17^ and found reduced VIN3-GFP RI>DD association at several of these loci (Figure S8C). This implicates VEL-mediated polymerization in promoting and maintaining VIN3 chromatin association with *FLC* and other loci in a PRC2-independent manner.

As introduced earlier, an emerging paradigm for the function of head-to-tail protein polymerization is the increase in local concentration of polymerizing proteins and their ligand binding sites, which enhances their binding avidity for low-affinity ligands (functional affinity).^6,8,31,32^ Because the head and tail interfaces of the VEL domain facilitates homotypic and heterotypic interactions between the VEL proteins, both types of interactions can theoretically contribute to promote VIN3 chromatin association in such a mechanism. We observed that VIN3-GFP VRN5VEL does not restore the VRN5-dependent interaction with PRC2, yet it still binds to *FLC* and other VIN3 target genes (Figure S8D) efficiently; thus, the properties of VIN3-VRN5VEL are sufficient for some but not for all outputs achieved by the VIN3 WT protein and its VEL domain. This highlights the complexities at play *in vivo*.

We decided to test whether a functionally relevant binding site—whose avidity for its chromatin ligand might be enhanced by the VEL-dependent polymerization—is present in the VIN3 protein itself. We turned to the tripartite PHD superdomain of VIN3: although this domain is an atypical PHD domain in that it exhibits no histone H3 tail binding activity, it has a weak affinity for negatively charged DNA or RNA polymers *in vitro*.^14^ We generated stable transgenic Arabidopsis plants carrying VIN3-GFP with a deletion of the entire PHD superdomain (ΔPHDsuper) in the *vin3* mutant background. The construct did not complement the *vin3* mutant in *FLC* vernalization time course experiments in two homozygous lines or in multiple other lines tested in the segregating T2 generation (Figure 4D, S9A, and S9B).

While VIN3 ΔPHDsuper maintained its interaction with PRC2 based on FIE immunoprecipitation in vernalized seedlings (Figure 4E), chromatin association with the *FLC* nucleation region was abolished (Figure 4F). This demonstrates that the VIN3 PHD superdomain is necessary for chromatin association and has chromatin or chromatin-associated ligands other than histone H3 tails. For VIN3-GFP RI>DD, the binding peak at the *FLC* nucleation region was much smaller than for VIN3-GFP WT but not entirely abolished (Figure 4C). This may reflect the weak chromatin affinity of VIN3 monomers, mediated by the PHD superdomain in the absence of the VIN3-mediated polymerization. We previously found an interaction between VIN3 and the transcriptional repressor VAL1, which serves as an assembly platform for co-transcriptional repressors and chromatin regulators.^17,33^ VAL1 binds to two RY motifs in the first *FLC* intron and could thus also provide a sequence-specific link to the *FLC* locus.^34^ However, while transgenic Arabidopsis plants carrying a point mutation in the first RY site (*FLC*-C585T) fail to nucleate H3K27me3, they still show an accumulation of H3K27me2 specifically in the nucleation region, indicating that VAL1 is only one of the multifactorial mechanisms that ensure targeting of VEL-PRC2 to this region.^17,34^ In agreement with this, we found that VIN3-GFP is still recruited to *FLC* in the C585T background (Figures S9C and S9D).

### VEL polymerization reinforces the chromatin association of VRN5-PRC2

We hypothesized that by directly interacting with VRN5, chromatin association of VIN3 would also reinforce the chromatin association of VRN5-PRC2. We thus decided to test whether VIN3 polymerization promotes the chromatin association of VRN5. A transgenic line carrying VRN5 tagged with mScarletI in which the *FLC* silencing defect of the *vrn5* mutant is complemented (Figure S9E) was crossed into the VIN3-GFP WT and VIN3-GFP RI>DD lines, respectively. VRN5-mScarletI protein levels were equal in both backgrounds (Figure S9F). In ChIP-qPCR experiments in vernalized seedlings, VRN5-mScarletI could be detected at the *FLC* nucleation region in VIN3-GFP WT but not in the VIN3-GFP RI>DD background (Figure 4G). Polymerization of the VIN3 VEL domain thus promotes not only chromatin association of VIN3 but also of VRN5-PRC2 to enable the deposition of H3K27me3. That the VRN5 VEL domain is not essential for H3K27me3 nucleation suggests that the association of the VIN3 polymer promotes H3K27me3 nucleation by other means in addition to the direct recruitment of VRN5 and PRC2, e.g., by binding other protein effectors such as VEL1.

### Role of VEL1 during cold-induced *FLC* silencing

Although VEL1 was shown to be genetically dispensable for cold-induced *FLC* silencing,^20^ VEL1 does associate with the *FLC* nucleation region, based on ChIP-qPCR and ChIP-seq experiments.^17^ To further test the role of VEL1 during cold-induced *FLC* silencing, we generated new *vel1* mutants in the Col-FRI background with CRISPR-Cas9. Using a sgRNA targeting the third exon of *VEL1* and screening for edited plants, we obtained two transgene-free homozygous lines carrying 718-bp (line #7-4) and 43-bp (line #9-2) deletions, respectively (Figures S10A and S10B). These *vel1* mutants—unlike *vin3* and *vrn5* mutants—did not show impaired *FLC* silencing during a vernalization time course (Figure S10C), consistent with previously published results for a *vel1* T-DNA mutant.^20^ We then crossed *vel1* #7-4 to VRN5-SYFP2 WT and ΔVEL lines to determine whether VEL1 function might contribute to the rescue of *FLC* silencing observed in VRN5 ΔVEL lines and analyzed *FLC* expression levels in homozygous plants. We observed impaired *FLC* silencing in VRN5 ΔVEL *vel1* but not in VRN5 WT *vel1*, suggesting that the presence of VEL1 can compensate for defects caused by VRN5 ΔVEL. However, the impaired *FLC* silencing observed in *vrn5* and *vin3* mutants and in VIN3-GFP RI>DD/ΔVEL lines indicates that VEL1 cannot compensate for all aspects of VIN3/VRN5 function during vernalization. It is possible that VIN3-VRN5VEL chromatin association (Figures 4B and S8D) may also be facilitated by interaction with VEL1.

## Discussion

Polymerization is one example of mechanisms that can achieve combinatorial protein inputs to promote the interaction between otherwise weakly interacting molecular components. Our findings suggest that polymerization via the VEL domain results in a high local concentration of VIN3, enhancing its avidity for chromatin by emergent multivalent interactions between VIN3 PHD superdomains and chromatin ligands (Figure 4H). The specific chromatin ligands of the PHD superdomain remain to be identified: unlike most PHD domains found in other proteins, our previous study using nuclear magnetic resonance (NMR) and ITC revealed that the PHD superdomain in VEL proteins is atypical in that it does not bind to histone tails.^14^ Interestingly, however, H2A was among the significant VIN3 interactors identified by native coIP-MS (Figure 1E), more specifically the histone variant H2A.W that is known to associate with H3K9me2-marked heterochromatin.^35^

Following the identification of *cis*-regulatory Polycomb response elements (PREs) in *Drosophila*, early models placed proteins with sequence-specific DNA-binding activity at PREs at the base of a linear Polycomb recruitment hierarchy.^36^ Our findings here fit into a more recently emerging complex picture where non-sequential and multifactorial protein interactions with the local chromatin environment, also shaped by transcriptional activity, give rise to genome-wide Polycomb silencing patterns.^37–40^ In what has been termed a “responsive model” for targeting Polycomb complexes, Polycomb samples along the chromatin for permissive chromatin states and accumulates at specific sites through positive feedback mechanisms.^41^ We propose that higher-order protein assemblies mediated by polymerization of VEL proteins constitute such a positive feedback mechanism. Bridged by VRN5, prolonged chromatin association of VIN3 can thus reinforce PRC2 chromatin association to facilitate H3K27me3 nucleation (Figure 4H). This is consistent with our previous observation that the *vin3* mutant, unlike *vrn5*, accumulates the precursor mark H3K27me2 in the nucleation region, which indicated that VIN3 is required to overcome the threshold from dimethylation to trimethylation at *FLC*, rather than facilitating PRC2 recruitment per se.^17^ Similarly, the accumulation of H3K27me2 at PRC2 target sites was observed in mammalian cell lines combining knockouts of different PRC2 accessory proteins.^42^

Overall, our findings for the VEL proteins have a striking resemblance to a polymerization network involving multiple Polycomb repressive complex 1 (PRC1) subunits that engage in a combination of heterotypic and homotypic interactions to promote transcriptional repression in *Drosophila*. These interactions are mediated by the head-to-tail co-polymerization of SAM domains of Polyhomeotic (Ph) and Sex combs on midleg (Scm) and are linked to the Pho-repressive complex (PhoRC) subunit Sfmbt, which undergoes heterotypic SAM interactions with Scm but is unable to homopolymerize.^43–45^ In Sfmbt, the polar residues in one of the SAM interfaces at positions corresponding to the apolar residues in Ph and SAM were proposed to explain the lack of homo-polymerization capacity.^43^ The DNA-binding activity of the PhoRC contributes to this polymerization-mediated hub to promote the nucleation of PRC1 complexes at target loci. Polymerization-disrupting mutations in the SAM domain of Ph do not alter Ph chromatin association at most genomic binding sites and have been predominantly linked to changes in longrange chromatin interactions.^46^ While the relationship between VEL polymerization and chromatin looping is currently still unknown, *FLC* alleles have been observed to cluster during vernalization, and this is impaired in *vrn2* and *vrn5* mutants.^47^

Like the SAM domain-dependent co-polymer,^48^ the *in vivo* composition and dynamics of the VEL protein assembly will depend on the affinities for all possible homotypic and heterotypic interactions between head and tail interfaces of VEL proteins. Specific amino acid residues in the polymerization interface contribute to this, observed here for attenuated polymerization of VIN3 when carrying I575 mutated to the threonine found at the corresponding position in the VRN5 VEL domain (Figure 3G). The threonine residue is widely conserved throughout angiosperm VRN5 orthologs (Figure 3F; see also Figure S7; Table S1), all predicted to be direct interactors of PRC2 based on the compact conformation of their PHDsuper and FNIII domains.^17^ By contrast, amino acids able to engage in hydrophobic interactions in the polymerization interface are prevalent in the corresponding position of angiosperm VIN3/VEL1 orthologs (Figure 3F), which are predicted to have a more open conformation of their PHD superdomain and FNIII domain, unable to confer PRC2 interaction.^17^ At least two homologs of VEL proteins, from each of the VRN5 and VIN3/VEL1-like subclasses, are present in these species, suggesting that the maintenance of VEL proteins with different PRC2 binding and polymerization properties throughout angiosperm evolution may be functionally important. Other amino acid differences in the polymerization interfaces, possibly associated with different posttranslational modifications *in vivo*, are likely to influence polymerization behavior to fine-tune the VEL polymerization network over evolutionary timescales and remain to be investigated in the future.

Overall, our work defines the combinatorial roles of VEL polymerization domains in maintaining the chromatin association of Polycomb proteins to enable the digital switch to the Polycomb silenced state, extending our mechanistic understanding of the principles not only underlying Polycomb switching but also eukaryotic gene regulation generally.

## Limitations Of The Study

The *in planta* analysis demonstrates that the PHD superdomain of VIN3 plays a role in chromatin association; however, the chromatin ligands of this domain still require further investigation. In our model of polymerization-mediated multivalent binding, we predict that the interaction between VIN3 monomers and chromatin has low affinity, which poses a particular challenge for testing putative chromatin ligands using traditional biochemistry techniques like coIP. Consistent with the proposed model, the *in vitro* interaction between the PHD superdomain and negatively charged nucleic acid is of very low affinity (dissociation constant [K_D_] of ~5 mM for double-stranded DNA [dsDNA]^14^); it remains to be tested whether this interaction is physiologically relevant. Our genetic analysis indicated complex redundancy between VEL1 and VIN3/VRN5 in the regulation of *FLC* expression. Future work will be required to elucidate when the VEL proteins can compensate for one another. Additionally, all experiments with transgenic Arabidopsis plants were performed using whole seedlings and therefore represent an average from many tissues. We cannot exclude that some of the proteins studied here have cell-type-specific and tissue-specific interaction partners and functions related to the deposition of H3K27me3.

## Resource Availability

### Lead contact

Further information and requests for resources and reagents should be directed to and will be fulfilled by the lead contact, Caroline Dean (caroline.dean@jic.ac.uk).

### Materials availability

The plasmids and transgenic plants generated in this study are available from the lead contact upon request.

## Star★Methods

### Key Resources Table

**Table:** 

REAGENT or RESOURCE	SOURCE	IDENTIFIER
Antibodies		
Anti-GFP (immuno blot)	Roche	Cat# 11814460001; RRID: AB_390913
Anti-GFP (ChIP)	Abcam	Cat# ab290; RRID: AB_303395
Anti-FIE	Agrisera	Cat# AS12 2616; RRID: AB_3676233
Anti-Actin	Agrisera	Cat# AS132640; RRID: AB_2722610
Anti-mScarlet 5F8 (ChIP)	Chromotek	Cat# 5F8; RRID: AB_2336064
Anti-mScarlet 6G6 (immuno blot)	Chromotek	Cat# 6G6; RRID: AB_2631395
Anti-H3K27me3	abcam	Cat# ab192985; RRID: AB_2650559
Goat anti-mouse HRP	Cytiva	Cat# NXA931; RRID: AB_772209
Goat anti-rabbit HRP	Cytiva	Cat# NA9340; RRID: AB_772191
Bacterial and virus strains		
*Escherichia coli* HST08	Takara	Cat# 636763
*Escherichia coli BL21(DE3)-R3-pRARE2*	Addgene	Cat# 26242
*Agrobacterium tumefaciens* GV3101 pMP90	Lab stock	N/A
*Agrobacterium tumefaciens* C58C1	Lab stock	N/A
Chemicals, peptides, and recombinant proteins		
Acetosyringone	Sigma	#D134406
EDTA-free Protease Inhibitor Cocktail	Roche	Cat# 04693159001
GFP-Trap	Chromotek	Cat# gta-k 20
Glycoblue	Invitrogen	Cat# AM9516
InFusion kit	Takara	Cat# 638945
LightCycler 480 SYBR Green I Master Mix	Roche	Cat# 04887352001
Percoll	Merck	Cat# P7828
Phenol solution saturated with 0.1 M Citrate	Merk Life Science UK Ltd	Cat# P4682
Phenol:Chlorophorm:isoamylalcohol	Sigma	Cat# P3803-100ML
PhosSTOP	Roche	Cat# 4906845001
Phusion Polymerase	New England Biolabs	Cat# M0530
Prestained Protein Ladder Broad Range	NEB	Cat# P7719
Protein A Agarose coated with salmon sperm DNA	Sigma-Aldrich	Cat# 16-157
Protein G Agarose coated with salmon sperm DNA	Sigma-Aldrich	Cat# 16-201
RNaseOUT Recombinant Ribonuclease Inhibitor	Invitrogen	Cat# 10777019
Silwet-77	BHGS	Cat# SILXXX000001
SuperScript IV Reverse Transcriptase	Invitrogen	Cat# 18090050
SuperSignal West Femto	ThermoFisher Scientifc	Cat# 34095
T4 Polynucleotide Kinase	NEB	Cat# M0201S
TURBO DNase	Invitrogen	Cat# AM2239
Critical commercial assays		
QIAprep Spin Miniprep Kit	Qiagen	Cat# 27104
Deposited data		
VIN3/VRN5-GFP WT & ΔVEL proteomics	Database: PRIDE	PXD048844
VIN3-GFP VRN5VEL proteomics	Database: PRIDE	PXD064199
SlimVar imaging data	Database: BioStudies	S-BIAD1233
Confocal microscopy data	Database: BioStudies	S-BIAD1249
Experimental models: Organisms/strains		
*Arabidopsis thaliana* accession *Col-FRI^SF2^*	Lee and Amasino^49^	N/A
*A. thaliana vin3-1 FRI*	Sung and Amasino^18^	N/A
*A. thaliana vrn5-8 FRI*	Franco-Echevarria et al.^17^	N/A
*A. thaliana* pVIN3::VIN3-GFP *vin3-1 FRI*	Fiedler et al.^11^	N/A
*A. thaliana* pVIN3::VIN3-GFP R556D I575D *vin3-1 FRI*	Fiedler et al.^11^	N/A
A. thaliana pVIN3::VIN3-GFP ΔVEL *vin3-1 FRI*	This paper	N/A
A. thaliana pVIN3::VIN3-GFP VRN5 VEL *vin3-1 FRI*	This paper	N/A
*A. thaliana* pVIN3::VIN3-GFP ΔPHDsuper *vin3-1 FRI*	This paper	N/A
*A. thaliana* pVRN5::VRN5-SYFP2 *vrn5-8 FRI*	This paper	N/A
*A. thaliana* pVRN5::VRN5-SYFP2 ΔVEL *vrn5-8 FRI*	This paper	N/A
*A. thaliana* pVRN5::VRN5-mScarletI *vrn5-8 FRI*	Payne-Dwyer et al.^26^	N/A
*A. thaliana vel1 #7-4/9-3 FRI*	This paper	N/A
Oligonucleotides		
Primers used in this study	This paper	Table S2
Recombinant DNA		
Plasmid: pCAMBIA 1300 p35S: Ω-GFP-VIN3	Fiedler et al.^11^	N/A
Plasmid: pCAMBIA 1300 p35S: Ω-GFP-VRN5	This paper	N/A
Plasmid: pCAMBIA 1300 p35S: Ω-mScarletI-VRN5	This paper	N/A
Plasmid: pCAMBIA 1300 p35S: Ω-mScarletI-VRN5 AVEL	This paper	N/A
Plasmid: pCAMBIA 1300 p35S: Ω-GFP-VIN3-VRN5VEL	This paper	N/A
Plasmid: pSLJ-promoterVIN3::VIN3-GFP	Fiedler et al.^11^	N/A
Plasmid: pSLJ-promoterVIN3::VIN3-GFP R556D I575D	Fiedler et al.^11^	N/A
Plasmid: pSLJ-promoterVIN3::VIN3-GFP ΔVEL	This paper	N/A
Plasmid: pSLJ-promoterVIN3::VIN3-GFP VRN5 VEL	This paper	N/A
Plasmid: pSLJ-promoterVIN3::VIN3-GFP ΔPHDsuper	This paper	N/A
Plasmid: pSLJ-promoterVRN5::VRN5-SYFP2	This paper	N/A
Plasmid: pSLJ-promoterVRN5::VRN5-SYFP2 AVEL	This paper	N/A
Plasmid: pKI1.1R	Addgene	Cat# 85808
Plasmid: pKI1.1R sgRNA-VEL1	This paper	N/A
Software and algorithms		
Arivis Vision4D ver. 4.1.0.	Zeiss	www.zeiss.com
GraphPad Prism 10	GraphPad	https://www.graphpad.com
MacVector	MacVector Inc	https://macvector.com
Microsoft Excel	Microsoft	https://www.microsoft.com
Proteome Discoverer 3.1/3.2	ThermoFisher Scientifc	https://www.thermofisher.com
Other		
Phytozome ver11	Joint Genome Institute (USA)	https://phytozome-next.jgi.doe.gov/

### Experimental Model And Study Participant Details

*Arabidopsis thaliana* plants were used in this study. All mutants and transgenics are homozygous for the indicated genotype and are in the Col-FRI background, which is Columbia (Col-0) with an introgression of the San Feliu-2 (SF2) FRIGIDA allele (FRI).^49^ The *vin3-1* FRI *(vin3)*^18^ and *vrn5-8* FRI (*vrn5*) mutants were previously described.^17^ The transgenic lines promoterVIN3:VIN3-eGFP/*vin3-4* FRI (VIN3-GFP #22)^34^, promoterVIN3:VIN3-eGFP/vin3-4 *vrn5-8* FRI (VIN3-GFP #22 *vrn5-8*)^17^, promoterVIN3:VIN3-eGFP/vin3-1 FRI (VIN3-GFP WT #3-8) and promoterVIN3:VIN3-eGFP R556D/I575D *vin3-1* FRI (VIN3-GFP RI>DD)^11^ were described previously. The previously transcribed construct *FLC*-C585T^34^ was transformed into *FLClean* FRI^50^ and then crossed to promoterVIN3:VIN3-GFP #22/vin3-4 FRI to generate VIN3-GFP *FLC*-C585T #36.

Seeds were surface sterilized with chlorine gas, sown on MS media plates without glucose (pH 5.7) and stratified at 4 °C in the dark for 2 days. For non-vernalized (before cold) conditions, seedlings were grown for 10 days in long-day conditions (16 h light, 8 h darkness, 20 °C). After this pre-growth, seedlings were vernalized for 6 weeks (8 h light, 16 h darkness, 5 °C) (6WT0). Vernalized seedlings were returned to before-cold conditions described above for 10 days (6WT10) for post-cold samples. For seed generation, seedlings were transferred to soil after vernalization and cultivated in a glasshouse with controlled 22°C 16 h day and 20°C 8 h night conditions.

### Method Details

#### Generation of transgenic *Arabidopsis* lines and CRISPR mutants

All primers used for cloning newly generated constructs are listed in Table S2.

The genomic pENTR promoterVIN3::VIN3-GFP construct^34^ was modified to make pENTR promoterVIN3::VIN3-GFPΔPHDsuper and pENTR promoterVIN3::VIN3-GFP ΔVEL by Quickchange, using Phusion DNA Polymerase (New England Biolabs). To generate pENTR promoterVIN3::VIN3-GFP VRN5VEL, the VRN5 VEL domain was amplified from pENTR promoterVRN5-SYFP2 and swapped into pENTR promoterVIN3-VIN3-GFP by restriction-free cloning.

To generate pENTR promoterVRN5::VRN5-SYFP2, the VRN5 genomic region including endogenous promoter and terminator were amplified from genomic Col-0 DNA and cloned into pENTR using In-Fusion cloning (TaKaRa). SYFP2 was then inserted with In-Fusion cloning, followed by the insertion of a 10 amino acid linker between VRN5 and SYFP2 with Quickchange PCR using Phusion DNA Polymerase (New England Biolabs). This wild-type construct was modified by Quickchange, using Phusion DNA polymerase, to generate pENTR promoterVRN5::VRN5-SYFP2 ΔVEL and pENTR promoterVRN5::VRN5-mScarletI^26^, respectively. All plasmids were verified by sequencing. The pENTR-constructs were transferred to the binary vector pSLJ-DEST based on pSLJ755I6 (PPT resistance) or, in the case of VRN5-mScarlet, to pSLJ-DEST based on pSLJ6991 (Hyg resistance) with LR reaction (Invitrogen) and then transformed into *vin3-1* FRI or *vrn5-8* FRI mutants mediated by Agrobacterium C58 using the floral dip method. Transgene copy number was determined in T1 transformants by IDna Genetics (Norwich Research Park).

To generate Arabidopsis *vel1* mutants, we employed the CRISPR/Cas9 plasmid pKI1.1R following the protocol described.^51^ Briefly, pKI1.1R plasmid (Addgene #85808) was linearized by incubating 1.5 μg of the plasmid with A*ar*I restriction enzyme for 7h, and then dephosphorylated using the alkaline phosphatase rAPid (Roche; 4898133001). A target-specific gRNA was designed using CRISPR-P 2.0 (http://crispr.hzau.edu.cn/CRISPR2) to target the third exon of *VEL1*. Oligonucleotides harbouring the gRNA target (sgRNA_VEL1_F and sgRNA_VEL1_R; Table S2) were hybridised by slow cooling down from 95–25°C and then phosphorylated using the T4 polynucleotide Kinase (NEB; M0201S). The digested plasmid and the hybridised oligonucleotides were ligated using the T4 ligase (NEB; M0202S) and then transformed in *Escherichia coli* HST08 competent cells.^51^ (p9128) The plasmid sequence carried by transformants were verified by Sanger sequencing. The plasmid was transferred to *Agrobacterium tumefaciens* C58C1 strain by electroporation for Agrobacterium-mediated transformation of Col-FRI plants. T_1_ plants carrying the construct were selected on MS media supplemented with 15 μg/ml of hygromycin. Homozygous T_2_ plants were then backcrossed to the parental genotype to remove the transgene.

#### RNA extraction and RT-qPCR

RNA was extracted as described,^34^ using acidic phenol followed by lithium chloride precipitation. RNA was DNase treated with Turbo DNA Free DNase, then transcribed into cDNA with SuperScript reverse transcriptase IV (both Life Technologies) with gene-specific reverse primers (Table S2). qPCR was performed using SYBRGreen Master Mix I on a LightCycler 480 II (both Roche) with primer pairs listed in Table S2.

##### Co-immunoprecipitation and immunoblotting

For co-immunoprecipitation (coIP) analysis followed by immunoblot analyses, total proteins were extracted from 2-3 aliquots of 3 g frozen ground Arabidopsis seedling tissue with IP buffer (50 mM Tris-HCl pH 7.5, 150 mM NaCl, 0.5% NP-40, 1% Triton-X, EDTA-free protease inhibitor cocktail [Roche]). Lysates were cleared by filtering through miracloth followed by centrifugation (6,000 g, 30 min, 4°C) and then incubated with GFP-Trap (Chromotek) or Protein G agarose beads combined with mScarlet antibody (5F8, Chromotek) for 4h. Immunoprecipitates were washed four times with IP buffer and eluted by boiling in 4x NuPAGE LDS sample buffer for 10 min. Input and coIP fractions were separated by polyacrylamide gel electrophoresis (SDS-PAGE) and blotted onto polyvinylidine difluoride (PVDF) membranes. Primary antibodies anti-GFP (11814460001, Roche), anti-FIE (AS12 2616, AgriSera) and anti-RFP/mScarlet (6G6, Chromotek) were diluted 1:1000, anti-Actin was diluted 1:5000 (AS132640, Agrisera). Secondary antibodies were HRP-coupled. Blots were washed with TBS containing 0.05% Tween-20 and developed with SuperSignal West Femto Maximum Sensitivity Substrate (Thermo Scientific).

##### Co-immunoprecipitation and mass spectrometry

For coIP followed by mass spectrometry, total proteins were extracted from 3 g of frozen ground Arabidopsis seedling tissue with the IP buffer described above, with the addition of PhosSTOP according to manufacturer instructions (4906845001, Roche). IP was performed as described above. Immunoprecipitates were eluted by boiling for 15 min in 20 mM Tris-HCl pH 8, 2% SDS. Proteins in the eluate were precipitated with chloroform/methanol (1:4) on ice for 30 min, the pellet was then washed twice with methanol and once with acetone before drying. Protein pellets were resuspended in 50 μl of 1.5% sodium deoxycholate (SDC; Merck) in 0.2 M EPPS-buffer (Merck), pH 8.5 and vortexed under heating. Cysteine residues were reduced with dithiothreitol, alkylated with iodoacetamide, and the proteins digested with trypsin in the SDC buffer according to standard procedures. After the digest, the SDC was precipitated by adjusting to 0.2% trifluoroacetic acid (TFA), and the clear supernatant subjected to C18 SPE using home-made stage tips with C18 Reprosil_pur 120, 5 μm (Dr Maisch, Germany). Aliquots were analysed by nanoLC-MS/MS on an Orbitrap Eclipse™ Tribrid™ mass spectrometer coupled to an UltiMate_®_ 3000 RSLCnano LC system (Thermo Fisher Scientific, Hemel Hempstead, UK). The samples were loaded onto a trap cartridge (PepMap™ Neo Trap Cartridge, C18, 5um, 0.3x5mm, Thermo) with 0.1% TFA at 15 μl min^-1^ for 3 min. The trap column was then switched in-line with the analytical column (Aurora Frontier TS, 60 cm nanoflow UHPLC column, ID 75 μm, reversed phase C18, 1.7 μm, 120 Å ; IonOpticks, Fitzroy, Australia) for separation at 55°C using the following gradient of solvents A (water, 0.1% formic acid) and B (80% acetonitrile, 0.1% formic acid) at a flow rate of 0.26 μl min^-1^: 0–3 min 1% B (parallel to trapping); 3–10 min increase B (curve 4) to 8%; 10–102 min linear increase B to 48; followed by a ramp to 99% B and re-equilibration to 0% B, for a total of 140 min runtime. Mass spectrometry data were acquired with the FAIMS device set to three compensation voltages (-35V, -50V, -65V) at standard resolution for 1.0 s each with the following MS settings in positive ion mode: OT resolution 120K, profile mode, mass range m/z 300–1600, normalized AGC target 100%, max inject time 50 ms; MS2 in IT Turbo mode: quadrupole isolation window 1 Da, charge states 2–5, threshold 1e,^4^ HCD CE = 30, AGC target standard, max. injection time dynamic, dynamic exclusion 1 count for 15 s with mass tolerance of ±10 ppm, one charge state per precursor only.

For VIN3-GFP/VRN5-SYFP2 and the VEL deletion lines, the mass spectrometry raw data were processed and quantified in Proteome Discoverer 3.1 (Thermo); all mentioned tools of the following workflow are nodes of the proprietary Proteome Discoverer (PD) software. The *A. thaliana* TAIR10 protein database (arabidopsis.org; 32785 entries) was modified by removing accessions AT4G30200.1, AT4G30200.3, and AT4G30200.4 corresponding to 3 versions of the VEL1 protein. Only AT4G30200.2 corresponding to the canonical version of VEL1 was left in the database for clearer search and quantification results. The database search including a decoy search was performed with Mascot Server 2.8.3 (Matrixscience, London; in house server) with a fragment tolerance of 0.5 Da, enzyme trypsin with 2 missed cleavages, variable modifications were oxidation (M), acetyl (Protein N-term), phosphorylation (STY), methylation/dimethylation/trimethylation (K); fixed modification carbamidomethyl (C). Validation in PD was then performed using Percolator based on q-values and FDR targets 0.01 (strict) and 0.05 (relaxed). The workflow included the Minora Feature Detector with min. trace length 7, S/N 3, PSM confidence high. The consensus workflow in the PD software was used to evaluate the peptide identifications and to measure the abundances of the peptides based on the LC-peak intensities. For identification, an FDR of 0.01 was used as strict threshold, and 0.05 as relaxed threshold.

For quantification, three replicates per condition were measured. In PD3.1, the following parameters were used for ratio calculation: normalisation on total peptide abundances, protein abundance-based ratio calculation using the top3 most abundant peptides, missing values imputation by low abundance resampling, hypothesis testing by t-test (background based), adjusted p-value calculation by BH-method. The results were exported into a Microsoft Excel table including data for protein abundances, ratios, p-values, number of peptides, protein coverage, the search identification score and other important values.

For VIN3-GFP VRN5VEL, the mass spectrometry raw data were processed and quantified in Proteome Discoverer 3.2 (Thermo) using the search engine CHIMERYS (MSAID, Munich, Germany) with the with the inferys_4.7.0_fragmentation prediction model, 0.3 Da precursor tolerance, variable modification oxidation (M), fixed modification carbamidomethyl, 1 missed cleavage, minimum peptide length 8. The workflows were similar to described above with protein abundance-based ratio calculation using the top5 most abundant peptides and missing values imputation by low abundance resampling.

Protein lists obtained for VIN3 and VRN5 wild-type proteins were filtered for interactors that were positively enriched in comparison to a Col-FRI non-transgenic control sample with an adjusted *p*-value ≤ 0.05. Enrichment ratios of interactors predicted to have a nuclear localization were log2-transformed for both WT and mutant samples to generate the heatmaps visualising the IP-MS results. The full mass spectrometry proteomics data have been deposited to the ProteomeXchange Consortium via the PRIDE partner repository with the dataset identifiers PXD048844 (doi: 10.6019/PXD048844) and PXD064199 (doi: 10.6019/PXD064199).

#### Chromatin immunoprecipitation (ChIP)

Histone ChIP was performed with 2 g of formaldehyde-crosslinked material as described previously^17^ with the following modifications: after nuclei extraction with Honda buffer, nuclei were layered on a Percoll density gradient (75%/40% Percoll in Honda) and extracted from the interface between these layers after centrifugation (7.000 x *g* in a swing bucket rotor for 30 min at 4 °C) prior to lysis of nuclei. Immunoprecipitation was performed with antibodies α-H3K27me3 (Abcam, ab192985) and α-H3 antibody (Abcam, ab1791), using 3 μg per IP reaction. Non-histone ChIP (VIN3/VRN5) was performed as described for GFP/YFP-tagged proteins.^17^ For lines with endogenous level VIN3 expression, each ChIP replicate was generated by pooling chromatin from three aliquots of 3 g of formaldehyde-crosslinked material for IP. Immunoprecipitation was performed with α-GFP (Abcam, ab290) using 3 μg per IP reaction.

#### Heterologous *Nicotiana benthamiana* transfections

The generation of *p35S:Ω-GFP-VIN3* was described previously.^11^ This plasmid was modified with seamless megaprimer cloning to generate *p35S:Ω-GFP-VRN5* and *p35S:Ω-mScarletI-VRN5* with the coding sequence of VRN5. Plasmids were transformed into *Agrobacterium tumefaciens* GV3101 using electroporation. Agrobacteria containing the desired construct at OD_600_ 0.05 were equally co-infiltrated with the silencing suppressor P19 into three-week-old *Nicotiana benthamiana* leaves. Confocal imaging of infiltrated epidermal leaf cells of *N. benthamiana* was performed on a Leica confocal Stellaris 8 microscope using a 63x/1.2 water objective and 4x zoom, excitation at 488 nm, detection at 507–542 nm for GFP and excitation at 561 nm, detection at 575–625 nm for mScarletI. Images were acquired 24 hr after infiltration with a laser speed of 600 Hz, a typical Z-step size of 4.7 μm and a pinhole size of 1 AU. The same settings were used at all imaging sets to allow direct comparison between constructs. The image analysis was performed in Arivis Vision4D ver. 4.1.0. (Zeiss). Firstly, the blob finder algorithm was applied to the GFP channel using a diameter value of 0.8 mm, a probability threshold of 50%, and a split sensitivity of 65%. Then, the blob finder algorithm was applied to the mScarletI channel using the same settings for diameter value, probability, and split sensitivity. Afterwards, the intersection between the output of the two blob finder operations was calculated. Finally, metrics such as volume were computed for the objects generated by each of the blob finder operations, as well as for their intersection. For the GFP channel-only analysis, an additional threshold was set: a minimum size of 0.03 μm^3^ and a sphericity (Mesh) of 0.6 during the analysis.

#### SlimVar microscopy and single-assembly analysis

The SlimVar technique detects rapidly diffusing assemblies, as small as single molecules, inside root tip nuclei. The microscope was employed in single-colour mode as described previously.^26^ Briefly, prepared seedlings were laid on a pad of MS growth media with 1% agarose on a standard slide, then coated with filtered MS media and sealed under #1.5 coverslips.

Individual nuclei within the outer three cell layers of the meristematic region of each root tip were identified in brightfield using a 100× NA 1.49 objective and centred in a region of interest no greater than 10 μm × 16 μm (190 × 300 pixel). Each nucleus of GFP- and SYFP2-labelled lines was illuminated rapidly at 3 kW cm^-2^ at an oblique angle of 60° with a collimated 488 nm or 514 nm laser respectively, and detected with a high performance sCMOS camera (Teledyne Prime95B) through a 500–550 nm or 525–575 nm emission filter respectively. The exposure time was 10 ms per frame, at a sampling rate of ~80 fps, with the sequence length sufficient to capture complete photobleaching down to single-molecule steps, typically ~1000 frames. Further independent measurements were taken with at least >3 nuclei per root and >3 roots per plate, for >3 independent growth and vernalization replicates (for details see Figure S3E).

In post-processing analysis, also following,^26^ diffraction-limited foci were extracted from each image sequence and connected into tracks that we identified with molecular assemblies. The stoichiometry of each track was estimated based on its initial fluorescent intensity, compared to that of the single label steps during late-stage photobleaching (see examples in Figures S3A and S3B). Stoichiometry distributions were collated from populations of tracked assemblies for each line and condition. The periodicity of each stoichiometry distribution was estimated from the most common peak-to-peak interval, in order to detect the presence of any regular structural subunits within assemblies.^26,52^ The periodicity analysis requires a minimum total number of tracks (approximately 14 tracks multiplied by the mean stoichiometry) for the intervals to be properly sampled to avoid missing peaks. To meet this requirement, the stoichiometry data are shown for each vernalisation cycle (the biological replicates shown in Figures 2A and 2B), but these replicates are aggregated for periodicity analysis for each line overall (Figures 2C and 2D). A global negative control was collated from simulated populations of uniform, random stoichiometry (grey dotted curves in Figures 2C and 2D); the distribution of intervals observed in this negative control was used as a proxy for p-value to reject the null hypothesis of no periodicity (corresponding to p=0.5 at 10% maximum interval fraction, p=0.05 at 19%, p=0.01 at 23% respectively).

Noting the previous observation that VRN5 assemblies above a threshold size of 10 molecules have a greater tendency to colocalize at the *FLC* locus as a result of vernalization,^26^ the proportion of assemblies above a stoichiometry of 10 was also determined for each population in this study. All pairwise comparisons of SlimVar stoichiometry data used the non-parametric Brunner-Munzel test.

##### SEC-MALS

Recombinant 6xHisLip-tagged VIN3_VEL_ (residues 500–603), either WT or I575T mutation were expressed and purified from *E. coli* and then used for SEC-MALS as previously described,^11^ with the following modification: SEC-MALS was performed using a Superose6 Increase 10/300 column.

#### Phylogenetic analysis

Protein sequences of VEL orthologs were from Phytozome ver11 (https://phytozome-next.jgi.doe.gov/). Alignments of protein sequences were done with MacVector (MacVector Inc) using the ClustalW algorithm.

### Quantification And Statistical Analysis

Statistical analyses for ChIP and gene expression analyses were performed using GraphPad Prism version 10.4.2. Details on number of replicates, error estimate, and significance cutoff can be found in the respective figure legends.

## Supplementary Material

Supplemental information can be found online at https://doi.org/10.1016/j.molcel.2025.08.002.

Supp Figs S1 - S10

Table S1

Table S2

## Data Availability

The MS proteomics data have been deposited to the ProteomeXchange Consortium via the PRIDE partner repository with the dataset identifiers PXD048844 (https://doi.org/10.6019/PXD048844) and PXD064199 (https://doi.org/10.6019/PXD064199). SlimVar microscopy data have been deposited to the BioStudies repository under the https://doi.org/10.6019/S-BIAD1233. All other microscopy data have been deposited to BioStudies under the https://doi.org/10.6019/S-BIAD1249. This paper does not report original code. Any additional information required to reanalyze the data reported in this paper is available from the lead contact upon request.

## Highlights

- VEL polymerization promotes multivalent chromatin association of Polycomb proteins
- VIN3 VEL polymerization is required for VIN3 assemblies and H3K27me3 nucleation
- VRN5 polymerization is dispensable unless VEL1 is absent
- VRN5 VEL is unable to replace VIN3 VEL but is required to link VIN3 to PRC2

## In brief

Schulten et al. show that VEL polymerization domains produce higher-order protein assemblies to maintain chromatin association of Polycomb proteins, enabling a stable epigenetic switch at the floral repressor gene *FLC* in *Arabidopsis*. This work highlights the importance of dynamic protein assemblies in regulating eukaryotic gene expression.
