# Supplementary material for: VEL-dependent polymerization maintains the chromatin association of Polycomb proteins for the switch to epigenetic silencing
Source: Mol Cell. Author manuscript; Available in PMC 2025 Oct 13. (PMC7618248; doi:10.1016/j.molcel.2025.08.002)
Supplement: Supp Figs S1 - S10 [file EMS209380-supplement-Supp_Figs_S1___S10.pdf]

Molecular Cell, Volume *85*

Supplemental information

VEL-dependent polymerization maintains the chromatin association of Polycomb proteins for the switch to epigenetic silencing

Anna Schulten, Geng-Jen Jang, Alex Payne-Dwyer, Marc Fiedler, Mathias L. Nielsen, Eduardo Mateo-Bonmatí, Mariann Bienz, Mark C. Leake, and Caroline Dean

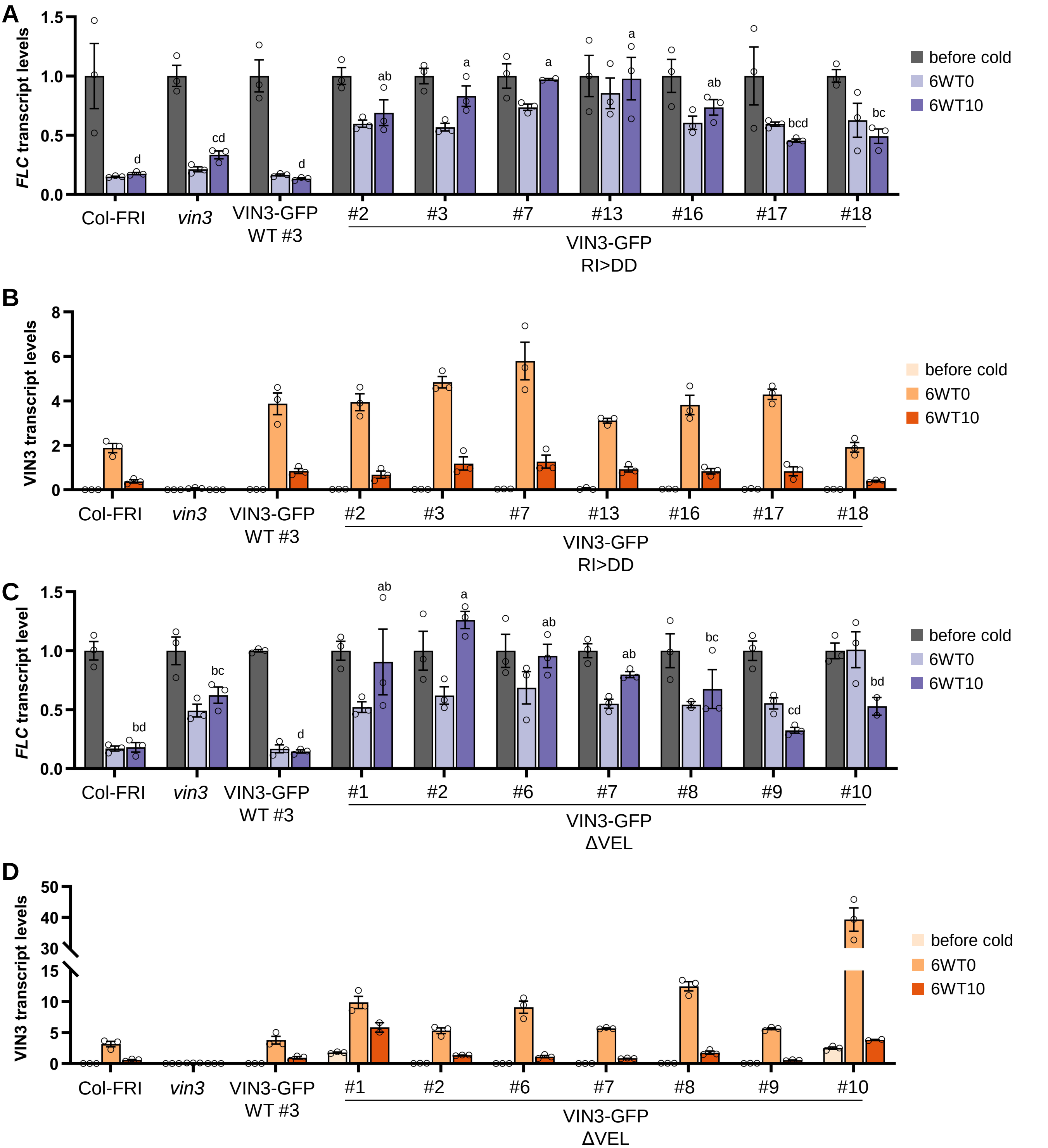

Fig. S1: Complementation analysis of VIN3-GFP RI>DD and ΔVEL mutant lines in the *vin3* background (related to Figure 1)

(A-D): RT-qPCR assays of *FLC* (A, C) and *VIN3* (B, D) transcript levels during a vernalization timecourse. RNA was extracted from homozygous plants (Col-FRI, *vin3*, VIN3-GFP WT #3) or from seven individual segregating T2 plant lines, grown on media supplemented with the herbicide PPT to select for resistant plants, before vernalization (before cold), at the end of a 6-week cold exposure (6WT0), or 10 days post-cold (6WT10). Data presented are relative to the geometric mean of *UBC* and *PP2A*. *FLC* transcript levels are normalized to *FLC* levels before the cold. Error bars represent standard deviations (n = 3 biological replicates). Different lowercase letters denote significant differences ($p < 0.05$) between means based on ANOVA with post-hoc Tukey's HSD.

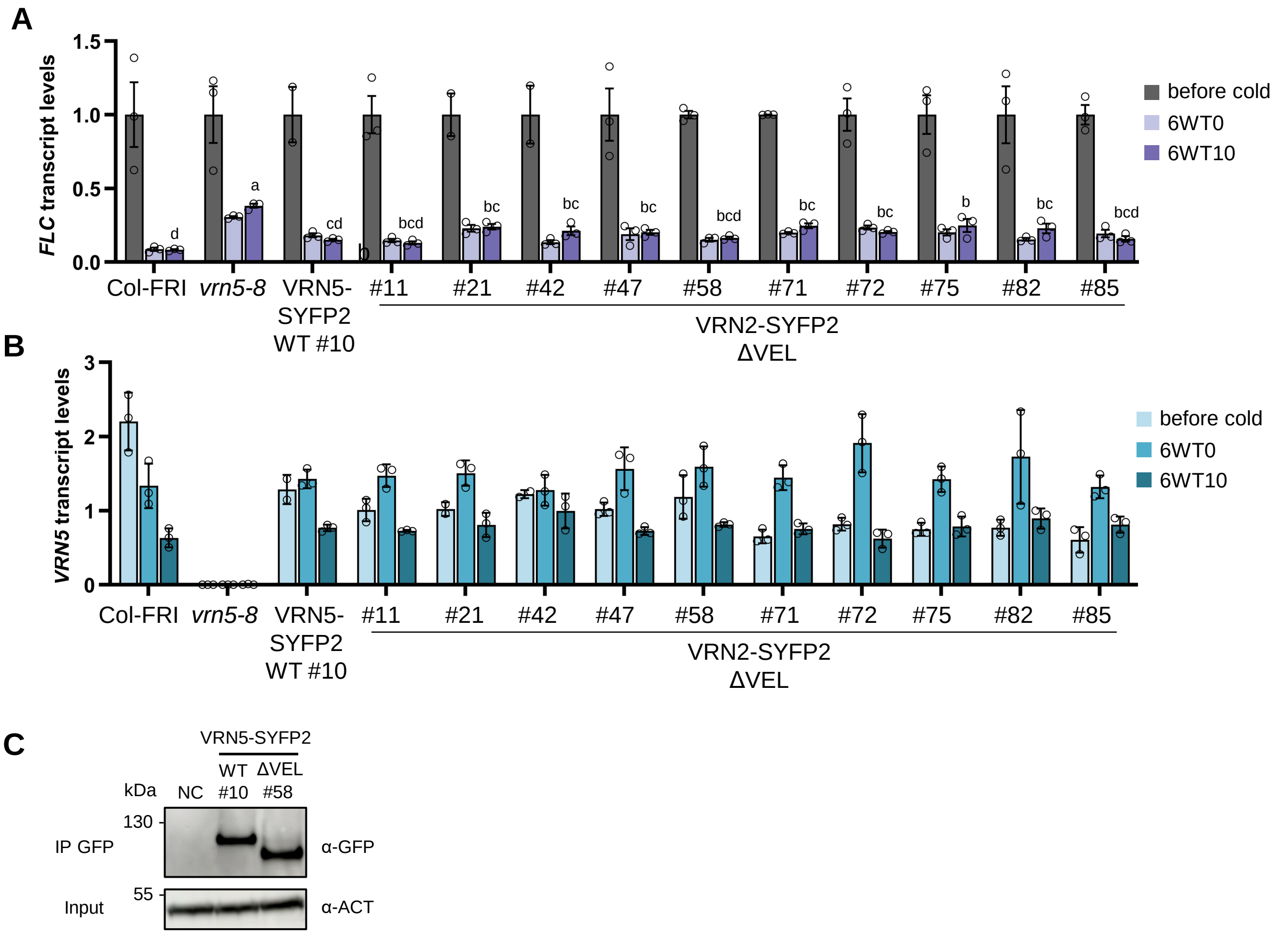

Fig. S2: Complementation analysis of VRN5-SYFP2 WT and ΔVEL mutant lines in the *vrn5* background (related to Figure 1)

(A, B) RT-qPCR assays of *FLC* (A) and VRN5 (B) transcript levels during a vernalization timecourse. RNA was extracted from homozygous plants (Col-FRI, *vrn5*, VRN5-SYFP2 WT #10) or from 10 individual segregating T2 plant lines (all single transgene insertion), grown on media supplemented with the herbicide PPT to select for resistant plants, before vernalization (before cold), at the end of a 6-week cold exposure (6WT0), or 10 days post-cold (6WT10). Data presented are relative to the geometric mean of *UBC* and *PP2A*. *FLC* transcript levels are normalized to *FLC* levels before the cold. Error bars represent standard deviations (n = 3 biological replicates), different lowercase letters denote significant differences ($p < 0.05$) between means based on ANOVA with post-hoc Tukey's HSD. (C) Immunoblots of α-GFP immunoprecipitates from extracts of vernalized plants (6 weeks) bearing the indicated VRN5-SYFP2 transgenes. Non-transgenic Col-FRI was used as a negative control (NC). Blots shown are a representative of three replicates.

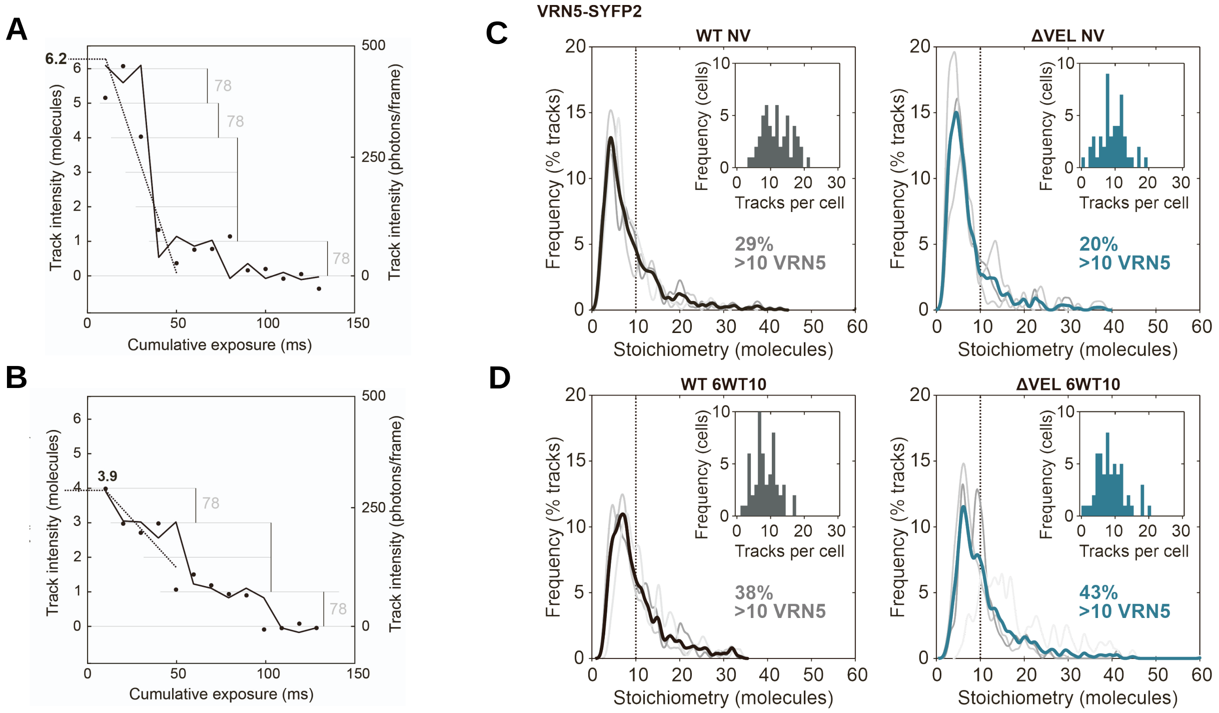

E

Timepoint	Line	Total nuclei	Tracks	Mean tracks per cell	± std.err.	Tracks % S>10	± std.err.
6WT0	VIN3-WT	86	1118	13	0.6	46%	4%
6WT0	VIN3-RIDD	34	567	16.7	0.7	29%	3%
NV	VRN5-WT	52	621	11.9	0.6	29%	1%
NV	VRN5-ΔVEL	47	444	9.4	0.5	20%	6%
6WT0	VRN5-WT	37	386	10.4	0.5	34%	4%
6WT0	VRN5-ΔVEL	43	394	9.2	0.5	48%	5%
6WT10	VRN5-WT	54	468	8.7	0.5	38%	5%
6WT10	VRN5-ΔVEL	55	492	8.9	0.5	43%	14%

Fig. S3: In vivo VEL protein assemblies (related to figure 2)

(A, B) Examples of molecular counting within tracked assemblies of fluorescent fusions during SlimVar. Traces are shown from photobleaching individual assemblies of (A) VIN3-WT-GFP and (B) VIN3-RI>DD-GFP at 6WT0. The raw intensity of each of the foci (black circles) was smoothed using an edge-preserving Chung-Kennedy filter (solid black lines). A consistent step height of 78 photons/frame corresponds to the characteristic molecular brightness of one GFP label, and therefore one labelled VIN3 molecule within the plant nucleus under the fixed SlimVar excitation and imaging settings. By back-extrapolating to the first frame prior to photobleaching (fit, dotted lines) and dividing by this characteristic molecular brightness, the total number of VIN3 molecules in the assembly (its stoichiometry, shown in bold) can be estimated to a higher precision than from individual frames or steps. (C, D) Distributions of stoichiometry (molecule number) of tracked assemblies of VRN5-SYFP2 WT and VRN5-SYFP2 ΔVEL at (C) non-vernalized NV or (D) post-vernalized 6WT10 conditions, in root nuclei of seedlings determined with SlimVar. Individual replicates (n = 3 experiments, with 9-18 nuclei each) are shown in grey, the coloured line indicates the mean distribution. Insets show the frequency of tracks per cell. (E) SlimVar statistics for data shown in main figure 2 and this supplemental figure. We predetermined a target sample size of >24 nuclei total per line per condition (coming from at least 3 experiments), powered to detect changes of <1 s.d. in each of the test variables (number of tracks, stoichiometry and periodicity) at a Bonferroni-adjusted significance level, here of $\alpha = 0.05/3 = 0.016$.

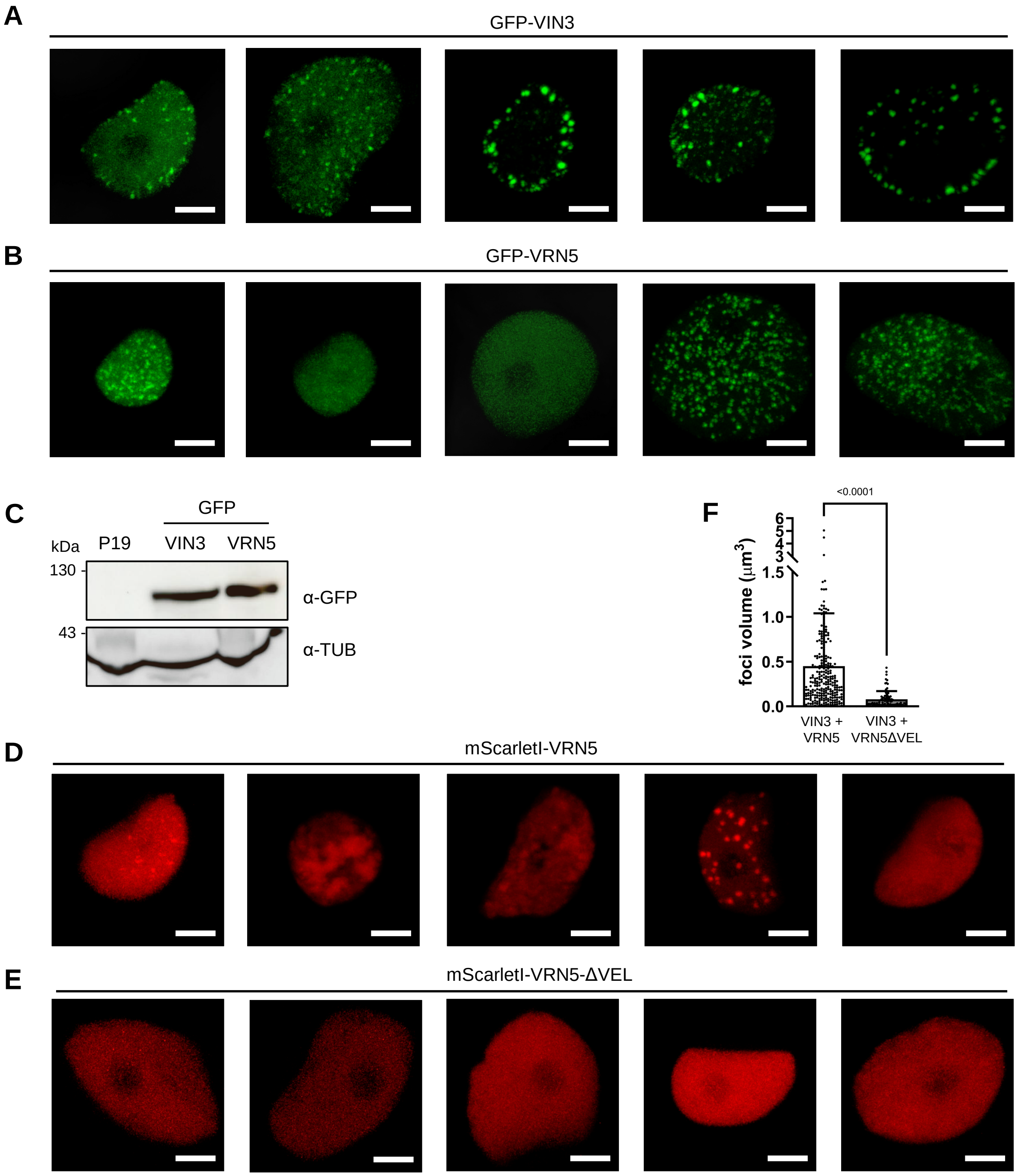

Fig. S4: Transient expression of VIN3 and VRN5 in *N. benthamiana* (related to Figure 2)

(A, B) Additional confocal images of epidermal leaf cell nuclei in *N. benthamiana,* transiently expressing GFP-VIN3 (A) or GFP-VRN5 (B), Scale bars: 5 µm (C) GFP immunoblots from extracts of *N. benthamiana* leaves in (A, B) transiently expressing GFP-VIN3 or GFP-VRN5 under a 35S promoter. P19 indicate leaves infiltrated with the P19 silencing suppressor only. α-Tubulin was used as a loading control. Blots shown are a representative of three replicates. (D, E) confocal images of epidermal leaf cell nuclei in *N. benthamiana,* transiently mScarletI-VRN5 (D) or mScarletI-VRN5 ΔVEL (E). Whole image brightness was adjusted based on the mean intensity of mScarlet in the nucleus. Scale bars: 5 µm. (F) Quantification of volume of foci that show co-localization of GFP-VIN3 with either mScarletI-VRN5 or mScarletI-VRN5 ΔVEL, shown in Fig. 2G. Error bars indicate standard deviations (n = 266 for VIN3 + VRN5, n = 83 for VIN3 + VRN5 ΔVEL). p-value indicates statistically significant difference based on two-tailed t-test.

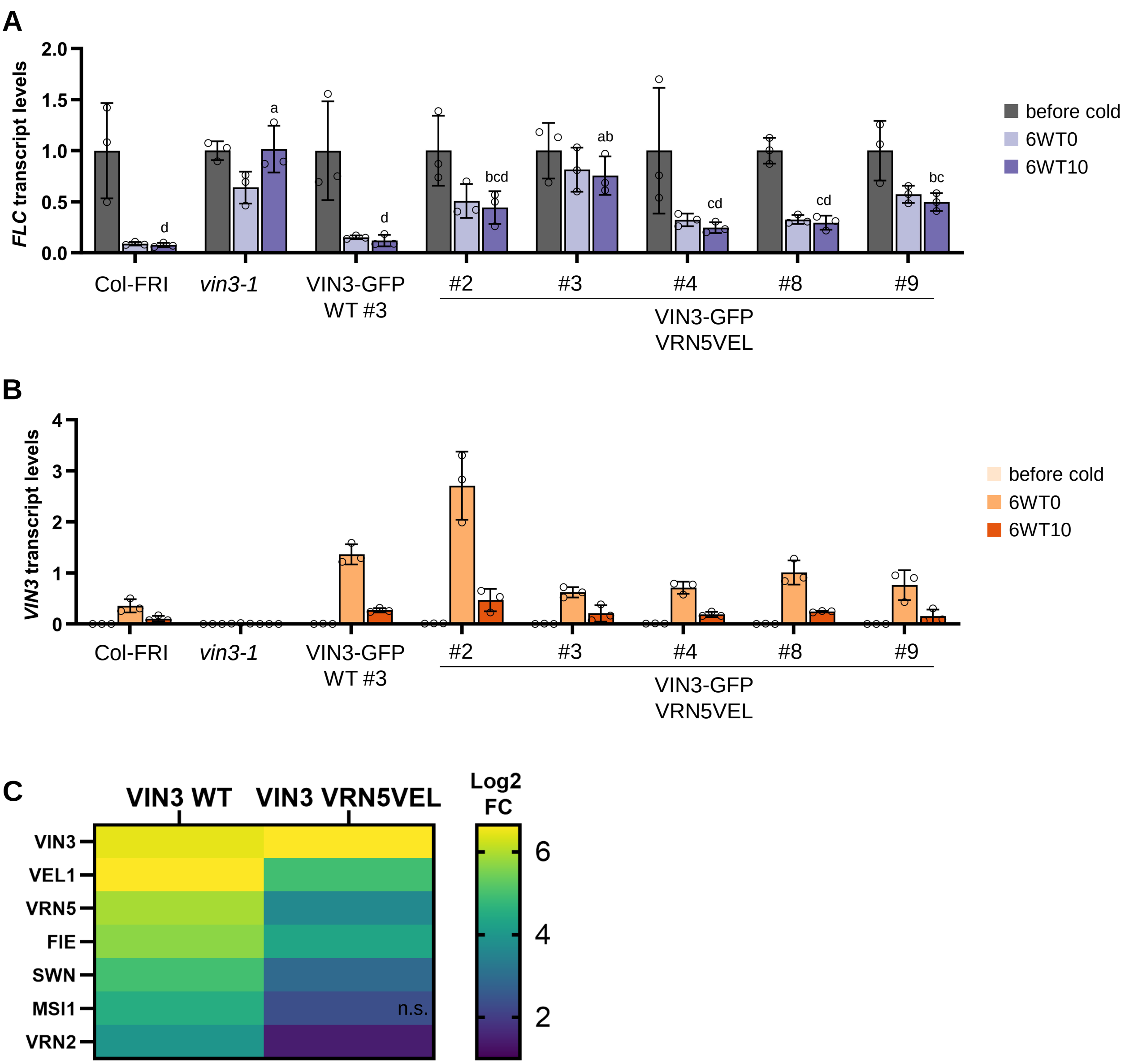

Fig. S5: Characterization of VIN3-GFP VRN5VEL mutant lines in the *vin3* background (related to Figure 3)

(A, B): RT-qPCR assays of *FLC* (A) and *VIN3* (B) transcript levels during a vernalization timecourse. RNA was extracted from homozygous plants (Col-FRI, *vin3*, VIN3-GFP WT #3) or from five individual segregating T2 plant lines, grown on media supplemented with the herbicide PPT to select for resistant plants, before vernalization (before cold), at the end of a 6-week cold exposure (6WT0), or 10 days post-cold (6WT10). Data presented are relative to the geometric mean of *UBC* and *PP2A*. *FLC* transcript levels are normalized to *FLC* levels before the cold. Error bars represent standard deviations (n = 3 biological replicates), different lowercase letters denote significant differences ($p < 0.05$) between means based on ANOVA with post-hoc Tukey's HSD. (C) Heatmap from IP-MS samples showing VEL proteins and PRC2 core subunits co-precipitating with VIN3-GFP WT and VIN3-GFP VRN5VEL baits in 6-weeks vernalized homozygous seedlings. log2 fold-change (FC) is in comparison to non-transgenic Col-FRI (adj. p-value ≤ 0.001, n = 3 biological replicates).

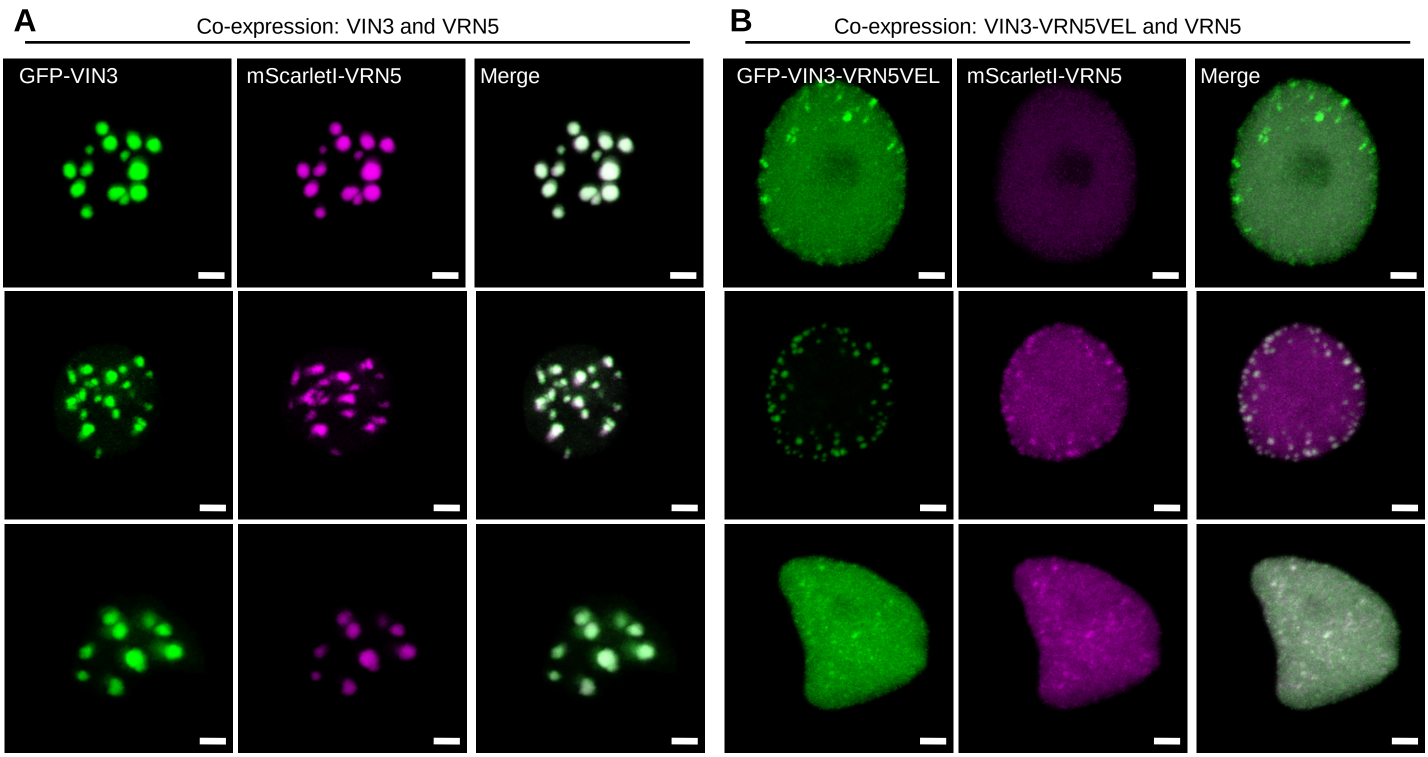

Fig. S6: Transient co-expression of VIN3 and VRN5 (related to Figure 3)

(A, B) Additional confocal images of epidermal leaf cell nuclei in *N. benthamiana,* transiently expressing GFP-VIN3 and mScarlet-VRN5 (A) or GFP-VIN3 VRN5VEL and mScarlet-VRN5 (B). Scale bars: 2 µm

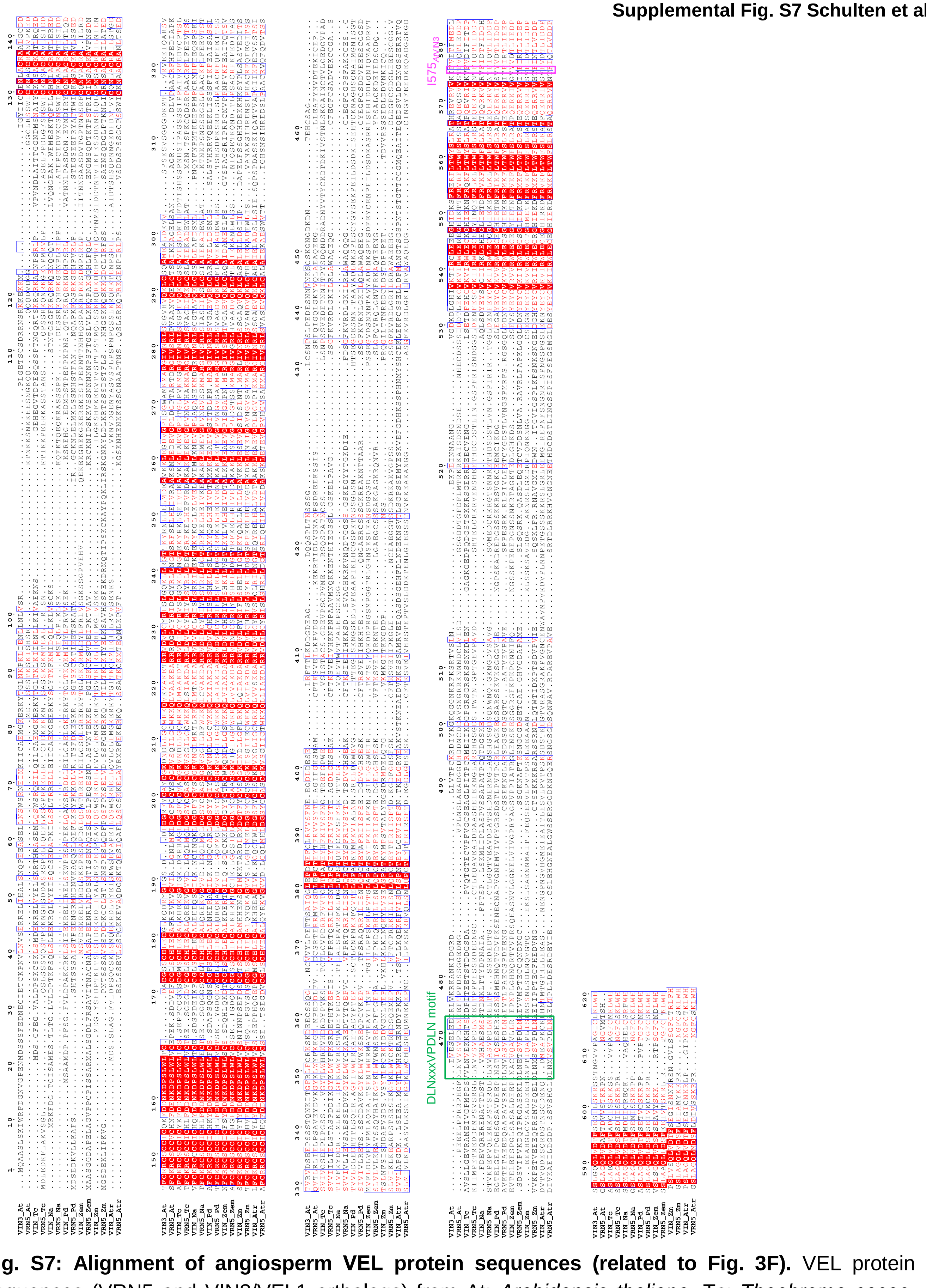

Fig. S7: Alignment of angiosperm VEL protein sequences (related to Fig. 3F). VEL protein sequences (VRN5 and VIN3/VEL1 orthologs) from At: *Arabidopsis thaliana*, Tc: *Theobroma cacao*, Na: *Nicotiana attenuata*, Pd: *Phoenix dactylifera*, Zem: *Zea mays*, Zm: *Zostera marina*, Atr: *Amborella trichopoda;* see Supplemental Table S1 for gene IDs. White in red boxes, invariant residues; red in blue frames, similar residues across species; green borders: DLNxxxVPDLN motif that defines VRN5 orthologs[14]; magenta borders: residue differing between VIN3/VEL1 orthologs (hydrophobic amino acids) and VRN5 orthologs (T).

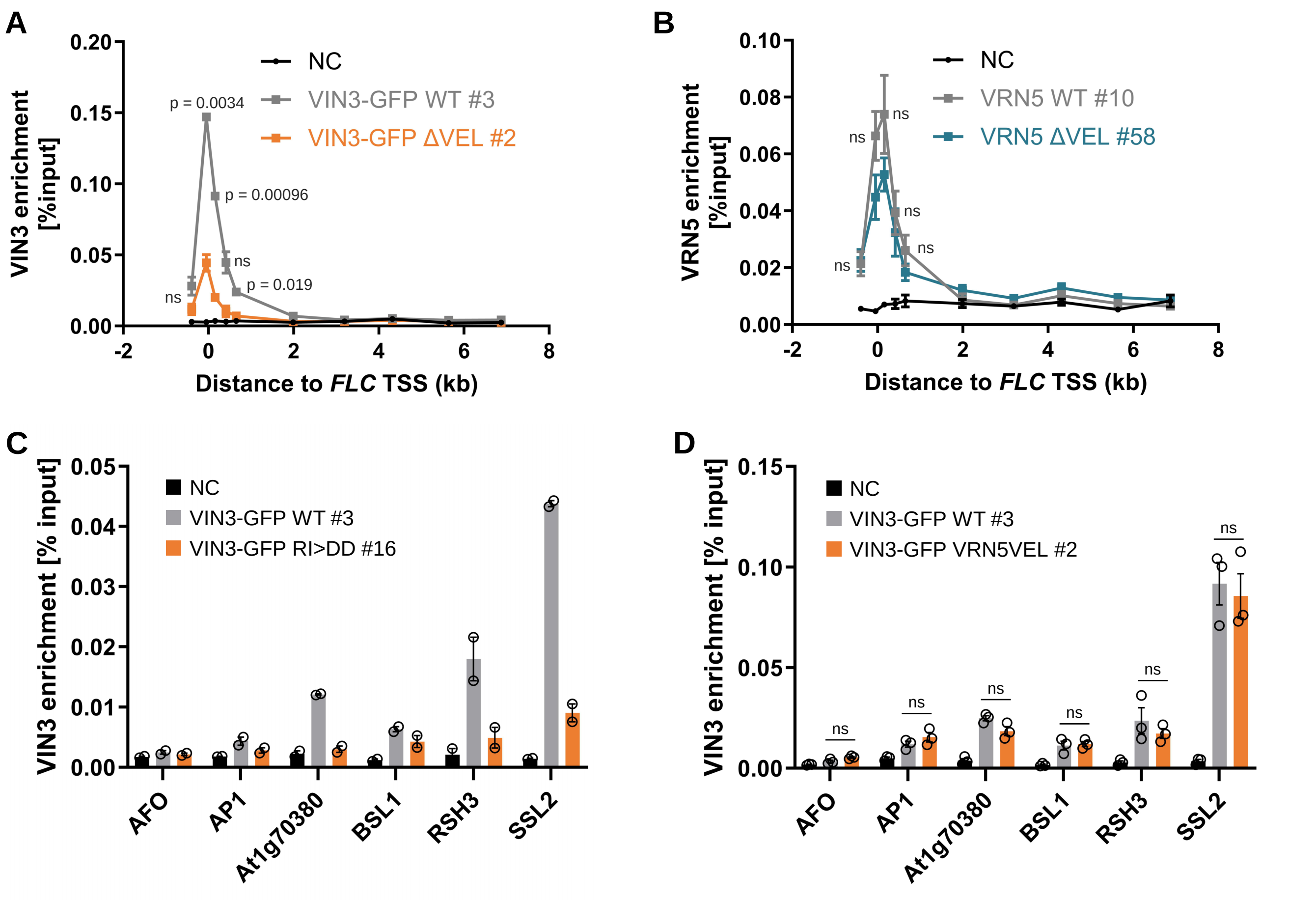

Fig. S8: Chromatin association of VIN3-GFP and VRN5-SYFP2 wild-type and mutant proteins as indicated (related to Figure 4)

(A, B) ChIP-qPCR showing enrichment of VIN3-GFP (A) and VRN5-SYFP2 (B) with wildtype or ΔVEL mutant protein across the *FLC* locus in seedlings vernalized for 6 weeks. Non-transgenic Col-FRI plants were used as a negative control sample (NC). (C, D) ChIP-qPCR showing enrichment of VIN3-GFP WT compared to VIN3-GFP RI>DD (C) or VIN3-GFP VRN5VEL (D) at VIN3 targets genes identified by ChIP-seq in seedlings vernalized for 6 weeks. All data are shown relative to an input control, error bars represent SEM (n = 2-6). Statistically significant differences between transgenic lines based on t-test are indicated by the p-value, ns: not significant ($p > 0.05$).

Supplemental Fig. S9 Schulten et al.

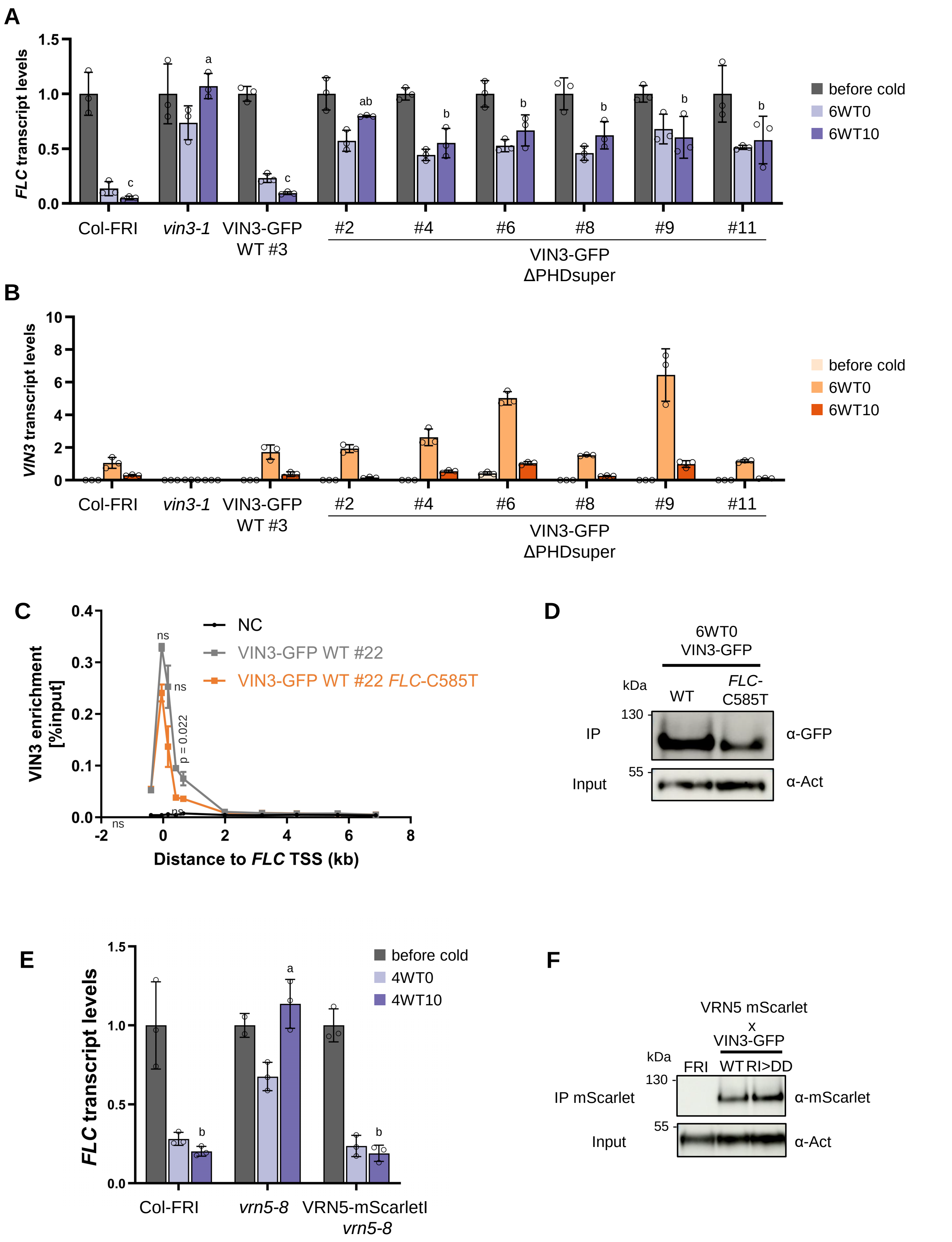

Fig. S9: Characterization of different VIN3/VRN5 transgenic lines as indicated (related to Figure 4)

(A, B) qRT-PCR assays of *FLC* (A) and *VIN3* (B) transcript levels in homozygous plants (Col-FRI, *vin3*, VIN3-GFP WT #3) or in six individual segregating T2 plant lines (VIN3-GFP ΔPHD super), grown on media supplemented with the herbicide PPT to select for resistant plants, before vernalization (before cold), at the end of a 6-week cold exposure (6WT0), or 10 days post-cold (6WT10). Data presented are relative to the geometric mean of *UBC* and *PP2A*. *FLC* transcript levels are normalized to *FLC* levels before the cold. Error bars represent standard deviations (n = 3 biological replicates), different lowercase letters denote significant differences ($p < 0.05$) between means based on ANOVA with post-hoc Tukey's HSD.

(C): ChIP-qPCR showing enrichment of VIN3-GFP across the *FLC* locus (either endogenous or *FLC*-C585T transgene with a point mutation in the first RY motif in intron 1 of *FLC)* in seedlings vernalized for 6 weeks. Statistically significant differences between transgenic lines based on t-test are indicated by the p-value, ns: not significant ($p > 0.05$). Note that the small reduction in VIN3-GFP enrichment in the *FLC*-C585T background is attributed to the slightly lower VIN3-GFP levels in this line in comparison to the parental line (panel B). Non-transgenic Col-FRI plants were used as a negative control sample (NC). Data are shown relative to an input control, error bars represent SEM (n = 2). (D) Immunoblots of α-GFP immunoprecipitates from extracts of vernalized plants bearing the VIN3-GFP transgene in the indicated background. Blots shown are a representative of two replicates.

(E) qRT-PCR assays of *FLC* transcript levels in homozygous plants (Col-FRI, *vrn5*, VRN5-mScarletI *vrn5*) before vernalization (before cold), at the end of a 4-week cold exposure (4WT0), or 10 days post-cold (4WT10). Data presented are relative to the geometric mean of *UBC* and *PP2A*. *FLC* transcript levels are normalized to *FLC* levels before the cold. Error bars represent standard deviations (n = 3 biological replicates), different lowercase letters denote significant differences ($p < 0.05$) between means based on ANOVA with post-hoc Tukey's HSD. (F) Immunoblots of α-mScarlet immunoprecipitates from extracts of vernalized plants bearing the VRN5-mScarletI transgene in the indicated transgene background (VIN3-GFP WT or VIN3-GFP RI>DD). Blots shown are a representative of two replicates.

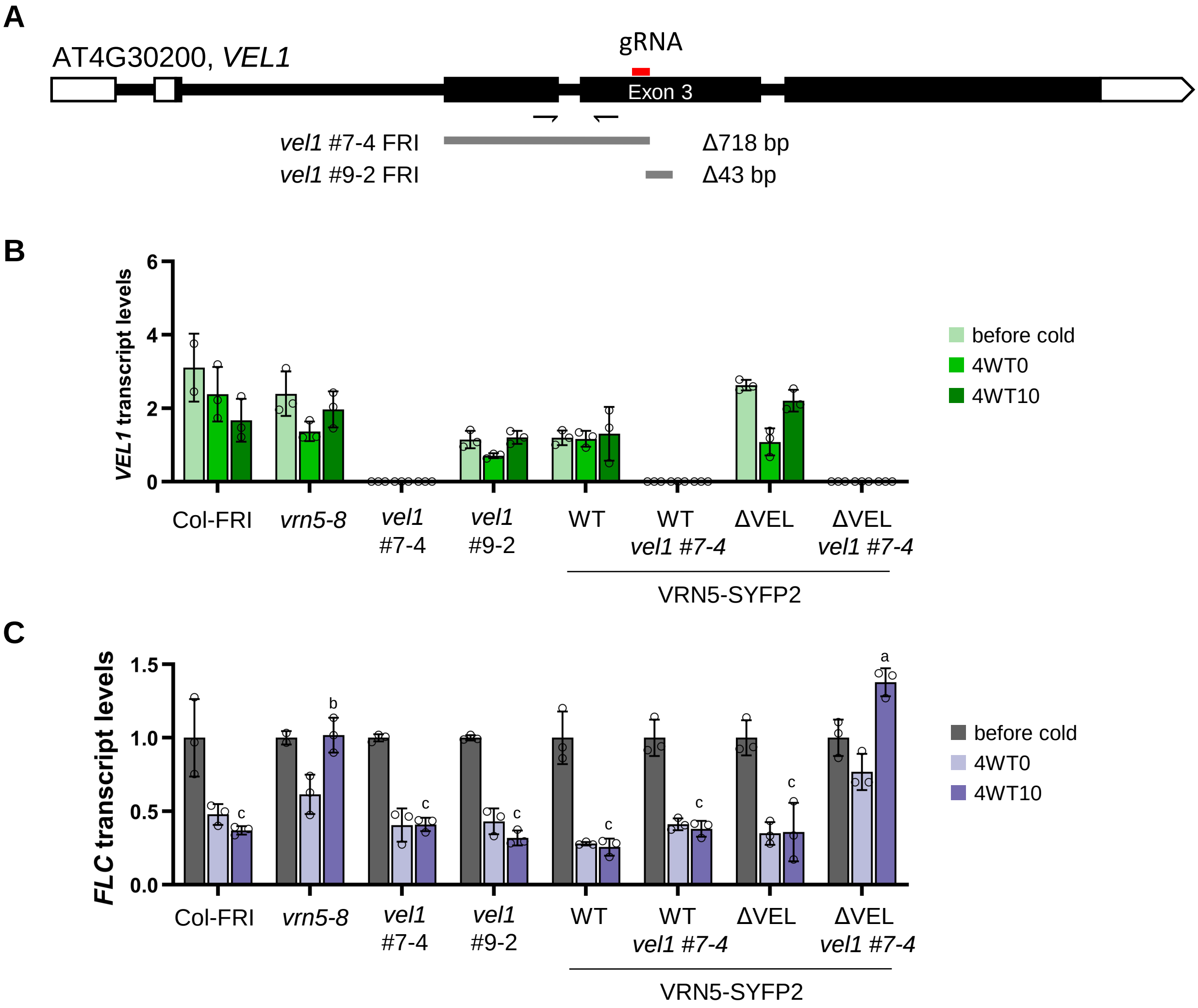

Fig. S10: *vel1* CRISPR alleles reveal that VEL1 mitigates defects caused by VRN5 ΔVEL during cold-induced *FLC* silencing (related to Figure 4)

(A) Schematic representation of CRISPR alleles in *VEL1.* Grey lines indicate genomic deletions in the corresponding *vel1* FRI mutant lines, red line indicates position of guide RNA (gRNA, not drawn to scale), harpoon arrows indicate primers used for qPCR in (B). (B, C) qRT-PCR assays of (B) *VEL1* deletion and (C) *FLC* transcript levels during a vernalization timecourse. RNA was extracted from homozygous plants before vernalization (before cold), at the end of a 4-week cold exposure (4WT0), or 10 days post-cold (4WT10). Data presented are relative to the geometric mean of *UBC* and *PP2A*. *FLC* transcript levels are normalized to *FLC* levels before the cold. Error bars represent standard deviations (n = 3 biological replicates), different lowercase letters denote significant differences ($p < 0.05$) between means based on ANOVA with post-hoc Tukey's HSD.
